# The Diagnosis and Management of Cardiometabolic Risk and Cardiometabolic Syndrome after Spinal Cord Injury

**DOI:** 10.3390/jpm12071088

**Published:** 2022-06-30

**Authors:** Gary J. Farkas, Adam M. Burton, David W. McMillan, Alicia Sneij, David R. Gater

**Affiliations:** 1 Department of Physical Medicine and Rehabilitation, School of Medicine, University of Miami Miller, Miami, FL 33136, USA; a.sneij@med.miami.edu (A.S.); dgater@miami.edu (D.R.G.J.); 2 Christine E. Lynn Rehabilitation Center for the Miami Project to Cure Paralysis, Miami, FL 33136, USA; dmcmillan@med.miami.edu; 3 School of Medicine, University of Miami Miller, Miami, FL 33136, USA; adam.burton@med.miami.edu; 4 The Miami Project to Cure Paralysis, School of Medicine, University of Miami Miller, Miami, FL 33136, USA

**Keywords:** spinal cord injury, cardiometabolic syndrome, cardiovascular disease, exercise, diet

## Abstract

Individuals with spinal cord injuries (SCI) commonly present with component risk factors for cardiometabolic risk and combined risk factors for cardiometabolic syndrome (CMS). These primary risk factors include obesity, dyslipidemia, dysglycemia/insulin resistance, and hypertension. Commonly referred to as “silent killers”, cardiometabolic risk and CMS increase the threat of cardiovascular disease, a leading cause of death after SCI. This narrative review will examine current data and the etiopathogenesis of cardiometabolic risk, CMS, and cardiovascular disease associated with SCI, focusing on pivotal research on cardiometabolic sequelae from the last five years. The review will also provide current diagnosis and surveillance criteria for cardiometabolic disorders after SCI, a novel obesity classification system based on percent total body fat, and lifestyle management strategies to improve cardiometabolic health.

## 1. Introduction

Spinal cord injury (SCI) is a life-altering medical condition resulting in the complete or partial loss of the afferent and efferent pathways within the spinal cord. The injury is characterized by a rapid onset of sublesional myopenia [1,2,3] and osteopenia [4] with subsequent accumulation in whole-body fat mass [5,6] two to seven months post-injury [7]. Increases in body fat, coupled with sedentary behavior/physical inactivity after SCI [8,9], predispose people with SCI to myriad health issues. Recent data provide evidence that cardiovascular disease has emerged as a leading cause of mortality in people with chronic SCI [10,11,12].

**Figure 1:**
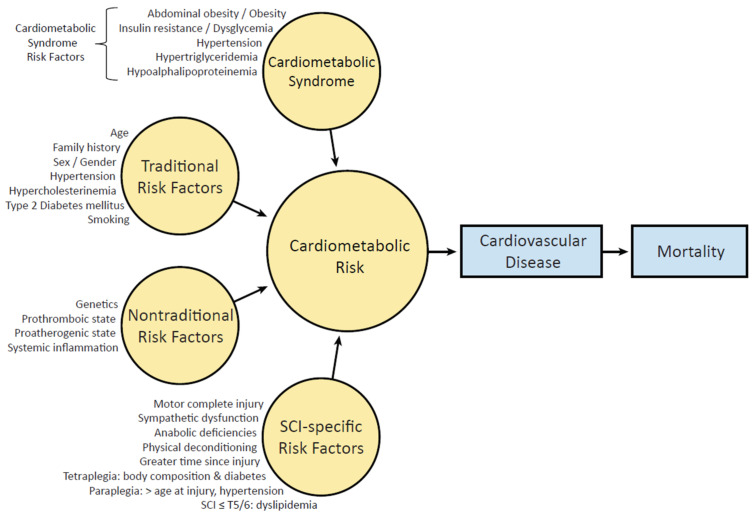
Interconnected component risk factors of cardiometabolic risk and cardiometabolic syndrome and their progression to cardiovascular disease and mortality.

**Figure 2:**
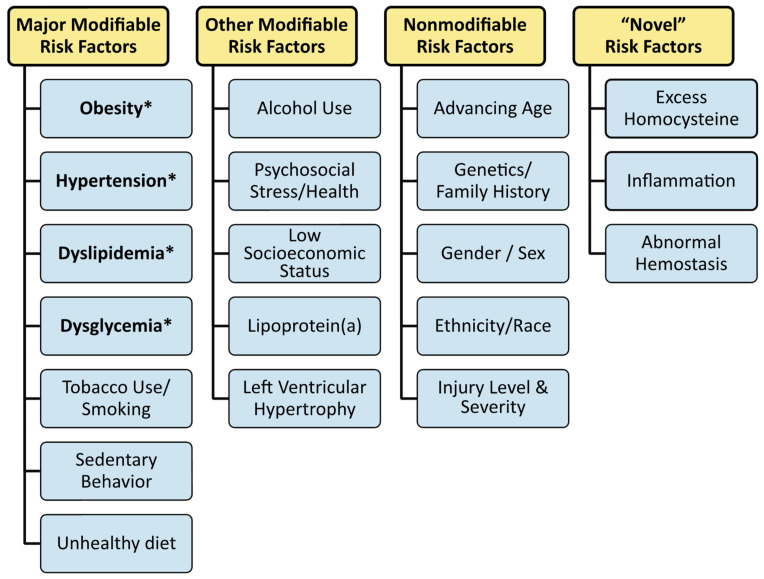
Modifiable and nonmodifiable risk factors for cardiometabolic risk. Component risk factors for cardiometabolic syndrome are marked with an asterisk (*).

A significant contributor to cardiovascular disease is cardiometabolic risk. When specific cardiometabolic risk factors co-manifest, they become a unique condition called cardiometabolic syndrome (CMS) that carries a risk comparable to type 2 diabetes mellitus and coronary heart disease. Cardiometabolic risk is the overall risk of cardiovascular disease resulting from the presence of CMS and traditional or nontraditional risk factors (Figure 1) [13]. Modifiable and nonmodifiable risk factors also increase cardiovascular disease risk (Figure 2) [14].

CMS (also called “syndrome X”, Reaven’s syndrome, insulin resistance syndrome, metabolic syndrome, and cardiometabolic disease) is a constellation of interrelated cardiometabolic risk factors (Figure 1). These risk factors, among others, appear to directly instigate the development of cardiovascular disease, cardiovascular mortality, and all-cause mortality [15]. The five most recognized definitions for the diagnosis and management of CMS are by the National Cholesterol Education Project Adult Treatment Panel III (NCEP ATP III) [16,17], the National Heart, Lung, and Blood Institute/American Heart Association (NHLBI/AHA) [18,19], the World Health Organization (WHO) [20], European Group for the Study of Insulin Resistance (EGIR) [21], and the International Diabetes Federation (IDF) (Table 1) [22,23]. Across the definitions, the specific component cardiometabolic risk factors are not in complete alignment, but the clustering of any group of these risk factors undoubtedly raises the threat of cardiovascular disease.

**Figure 3:**
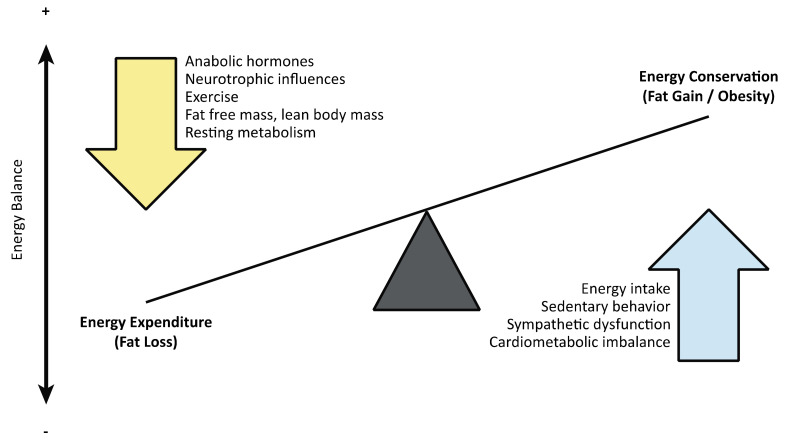
The relationship between energy expenditure and intake and the components influencing them following a spinal cord injury.

The most widely used component CMS risk factors are dyslipidemia, hypertension, dysglycemia/insulin resistance, and obesity. These risk factors, however, are not equally responsible for the development of CMS. CMS and its component risk factors are strongly attributed to obesity [13]. Obesity develops from a positive energy balance where total daily energy intake exceeds total daily energy expenditure (Figure 3) [27,28,29,30,31,32,33,34], thus making people with SCI susceptible to this risk factor and CMS [35].

In this narrative review, we provide the latest evidence on and the etiopathogenesis of cardiometabolic risk after SCI. We report on the primary overlapping cardiometabolic risk and CMS component risk factors (obesity, dyslipidemia, hypertension, and dysglycemia/insulin resistance) and their culminating threat, cardiovascular disease. The review examines the diagnosis and management of cardiometabolic risk after SCI, including a novel obesity classification system based on percent body fat and traditional obesity cutoff values. Furthermore, this review will focus on studies within the last five years with reference to seminal literature on cardiometabolic morbidities that helped guide the current population-specific identification and management systems used today.

## 2. Mechanisms Leading to Cardiometabolic Risk

**Figure 4:**
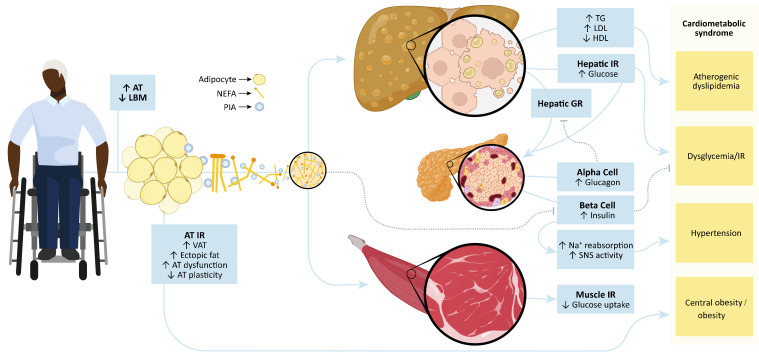
Spinal cord injury (SCI) morbidity presented as a continuum from the onset of neurogenic obesity to the development of cardiometabolic syndrome. SCI results in neurogenic obesity through the loss of metabolically active lean body mass (LBM) and a concurrent accumulation of adipose tissue (AT). Obesity-induced hypoxia results in the dysregulation of AT, marked by a loss of AT plasticity and the secretion of non-esterified free fatty acids (NEFA) and proinflammatory adipokines (PIA). NEFA enter peripheral circulation, resulting in visceral (VAT) and ectopic fat deposition, thereby promoting systemic insulin resistance (IR). NEFA deposition in the liver stimulates increased glucose production and hepatic IR. Hepatic NEFA accumulation also promotes atherogenic dyslipidemia through triglyceride (TG) lipogenesis and the increased and decreased production of LDL- and HDL-cholesterol, respectively. NEFA deposition also occurs in the nearby pancreas, inducing β-cell dysfunction by lipotoxicity and dysglycemia/diabetes. NEFA storage in the liver also promotes hepatic glucagon resistance (GR) and hyper-aminoacidemia that stimulates glucagon secretion to compensate for hepatic GR. Hyperglucagonemia facilitates increased hepatic glucose release. In skeletal muscle, increased NEFA deposition promotes IR, inhibiting insulin-mediated glucose uptake. Overall, the systemic state of IR results in hyperinsulinemia. Hyperinsulinemia may increase sodium (Na^+^) reabsorption and sympathetic nervous system activity above the level of SCI, contributing to hypertension. PIA alter signaling pathways contributing to atherogenic dyslipidemia, hypertension, and insulin resistance/dysglycemia environment. Collectively, when these metabolic morbidities co-manifest, they present as cardiometabolic syndrome. Arrows represent stimulation/enhancement, flat ends demonstrate inhibition/repression, and dashed lines represent a progressive decrease in a pathway. Caption adapted from [35].

Obesity manifests as the excessive accumulation of whole-body adipose tissue or whole-body fat. However, simply attributing cardiometabolic risk to merely an excessive amount of adipose tissue is an oversimplification. The *dysregulation* of adipose tissue with obesity is considered the actual origin of cardiometabolic comorbidities (Figure 4). Sakers et al. [36] recently argued that obesity-induced deleterious health outcomes originate not simply from an excessive amount of adipose tissue but from the weakened ability of the tissue to respond to physiological changes. Thus, with obesity, adipose tissue loses its plasticity and the ability to respond to physiological cues to maintain homeostasis. Mechanistically, excess adipose tissue leads to a state of adipose tissue hypoxia, a decrease in energy balance nutrient-buffering, and a loss of adipocyte mitosis. This hypoxic state results in insulin resistance, inflammation, and adipocyte apoptosis coupled with the uninhibited secretion of lipids (Figure 4) [36]. The excessive release of non-esterified free fatty acids (NEFA) from adipose tissue contributes to the accretion of ectopic lipids in locations other than adipose tissue [37,38,39]. Ectopic lipid accumulation in the liver [40] and muscle [41] predisposes people to insulin resistance and dyslipidemia pathogenesis [42], pathologies commonly reported after SCI [35,43,44,45].

Both obesity and CMS are also associated with a state of chronic, systemic inflammation. Some researchers hypothesize that this inflammatory state may underlie or exacerbate cardiometabolic risk [46,47]. Individuals with obesity present with adipose tissue that exhibits abnormal production and secretion of biologically active molecules, such as inflammatory adipokines and hemostasis-modulating compounds [48]. Adipocytes and the heterogeneous cells of the stromal vascular fraction secrete agents that modulate cardiometabolic profiles by altering homeostatic signaling cascades (Figure 4) [28,35,49]. Notable proinflammatory agents that directly affect signaling pathways related to insulin resistance, dyslipidemia, and/or vascular dysfunction/hypertension include tumor necrosis factor-α (TNF-α), interleukin-6 (IL-6), interleukin-1β (IL-1β), and monocyte chemoattractant protein-1 (MCP-1) [28,35]. TNF-α and IL-6 are implicated in dyslipidemia through the secretion of NEFA from visceral fat lipolysis. Excessive NEFA production increases hepatic production of apolipoprotein-B, low-density lipoprotein cholesterol (LDL-C), and very-low-density lipoprotein cholesterol. In contrast, there is a decrease in apolipoprotein-A output, reducing high-density lipoprotein cholesterol (HDL-C) (Figure 4). TNF-α and IL-6 suppress insulin receptor substrates 1 (IRS-1) and 2 (IRS-2) and glucose transporter-4 (GLUT-4) while upregulating suppressor of cytokine signaling-3, resulting in insulin resistance [35,50,51]. TNF-α, IL-6, and IL-1β activate nuclear factor kappa-light-chain- enhancer of activated β cells (NFκβ), which further blocks phosphorylation of IRS-1 and IRS-2, limiting the phosphoinositide 3-kinase (PI3K) cascade required for GLUT-4 migration to the cellular membrane [52]. Furthermore, TNF-α, IL-1β, and NFκβ induce pancreatic b-cell apoptosis in the advanced stages of type 2 diabetes mellitus, reducing the endogenous production of insulin [53]. The hemostatic agent plasminogen activator inhibitor-1 inhibits fibrinolysis, creating an atherosclerotic environment and endothelial dysfunction by preventing plasmin activity. Endothelial dysfunction and increased arterial stiffness result in the release of vascular cellular adhesion molecule-1, intercellular adhesion molecule-1, and MCP-1. MCP-1 leads to the migration and infiltration of circulating monocytes that differentiate into type II proinflammatory macrophages. These macrophages continue to secrete TNF-α and IL-6 into the local and systemic environment. People with and without SCI presenting with CMS component risk factors often present with a prothrombotic and proinflammatory environment [28,35]. These findings support the notion that obesity, mediated by an “adipose tissue disease” [54], is the driving factor of cardiometabolic risk.

## 3. Obesity after SCI

Sedentary activity and/or positive energy balance are the two most common drivers of obesity for individuals without SCI. However, for individuals with SCI, sedentary activity and/or positive energy balance insufficiently characterize the unique pathophysiology that results in obesity. In 2018, Farkas and Gater [28] first presented the term “neurogenic obesity” in a narrative review of chronic, low-grade systemic inflammation in people with longstanding SCI. The authors characterized not just sarcopenic obesity due to muscle atrophy (“obligatory sarcopenia”) but also paralysis-induced neurogenic osteoporosis, loss of neurotropic influences, anabolic deficiency, sympathetic dysfunction, and blunted satiety associated with SCI that profoundly reduces whole-body energy expenditure [28,35]. These SCI-induced changes create an obesogenic environment that, coupled with sedentary activity and positive energy balance, results in a significant accumulation of body fat (Figure 3).

An increased amount of body fat characterizes obesity, historically defined by Heyward with the American Society of Exercise Physiologists as a total percent body fat (%BF) > 22% in men and >35% in women [55,56]. This definition of obesity is seldom used, and few studies measure %BF in people with and without SCI. The infrequent use of %BF thresholds underscores the difficulty in measuring body composition. Specifically, measurements require specialized equipment, technical skill, and a considerable amount of time. Because of the complexity of measuring total %BF, authoritative professional organizations have developed surrogate anthropometric measures to quantify obesity.

Body mass index (BMI) and waist circumference (WC) are the two most common anthropometric measures used to quantify obesity, and both are problematic for use in the SCI population. Both the World Health Organization (WHO) [57] and the Centers for Disease Control and Prevention (CDC) [58] use BMI to define obesity. BMI is a simple index of weight-for-height and is defined as a person’s weight in kilograms divided by the square of height in meters (kg/m^2^). Table 2 describes the four BMI categories and one subcategory that classifies obesity with three levels. In addition to BMI, WC is used to determine obesity, specifically central or abdominal obesity, at the level of the umbilicus. In a Consensus Statement from the Association for Weight Management and Obesity Prevention, the North American Association for the Study of Obesity of the Obesity Society, the American Society for Nutrition, and the American Diabetes Association, Klein et al. [59] proposed using a WC > 102 cm in men and >88 cm in women to define obesity in people without SCI (Table 2). WC has not been validated in people with SCI and is a suboptimal surrogate of obesity in this population, given the varying neurological levels and completeness of abdominal muscle paralysis [45,60]. Similarly, BMI understates obesity in people with SCI due to the reduction of fat-free mass reflecting the obligatory sarcopenia, osteopenia, and reduced total body water associated with paralysis. In fact, BMI does not consider the *composition* of total body weight compared to obesity, defined by the anatomic estimate of adipose tissue load. Despite the profound limitations of these anthropometrics, Silveira et al. [61] reported that BMI, with its standard definitions, was the most commonly used method to quantify and describe obesity after SCI.

Several studies have developed SCI-specific BMI and WC obesity cutoffs and assessed their utility in identifying cardiometabolic risk (Table 3). Laughton et al. [62], in 77 community-dwelling Canadian adults with chronic SCI, developed the most widely used population-specific BMI using piecewise linear regression and a receiver-operator characteristic (ROC) curve. Based on total %BF and C-reactive protein, the authors derived a BMI cutoff > 22 kg/m^2^ to define obesity [62]. Ayas et al. [63] developed a higher cutoff using the median BMI of 25.3 kg/m^2^ to define obesity in habitual snorers with SCI in the United States (US). However, most other studies have developed similar or lower cutoffs to Laughton et al. [62]. Yun et al. [64] established population-specific cutoffs for BMI and WC utilizing ROC curves and the Youden index in Korean men with motor complete SCI compared to matched controls. The authors identified an SCI-specific BMI of 20.2 kg/m^2^ and a WC of 81.3 cm compared to 22.5 kg/m^2^ and 85.5 cm in the controls [64]. Shin et al. [65], using the area under the ROC curve, assessed BMI’s validity in diagnosing CMS in 157 Korean individuals with chronic SCI. The authors found that a CMS diagnosis was associated with a BMI cutoff of 22.8 kg/m^2^ [65]. In 74 Japanese men with SCI, Inayama et al. [66] used nonlinear regression to compute a WC of > 81.3 cm and a BMI of >22.5 to identify visceral fat area > 100 cm^2^ (a frequently cited obesity cutoff for visceral fat [61,67,68]). Other authors only examined WC, including Ravensbergen et al. [69] reported that adverse cardiovascular disease risk was identified as a WC ≥ 94 cm in individuals with SCI utilizing ROC curves. Using linear regression, Sumrell et al. [70] developed an SCI-specific WC of 86.5 cm in motor complete SCI. Using the cutoff by Sumrell et al. [70], Gill and colleagues [71] reported that 36% of participants with motor complete injuries were classified as obese compared to 3% when using a WC > 102 cm. When pooling SCI-specific anthropometric values from these studies, >23.3 kg/m^2^ and >83.9 cm represent a weighted threshold for BMI and WC, respectively (Table 3). These studies seem to support the harmony regarding an SCI-specific BMI threshold; however, less consensus exists concerning WC. Further studies are also needed to validate the current metrics

To date, only one study has compared published cutoff values in the SCI population. In veterans with SCI, Yahio et al. [72] tested three published BMI cutoffs: WHO (30 kg/m^2^), Ayas et al. (25.3 kg/m^2^) [63], and Laughton et al. (22 kg/m^2^) [62]. These cutoffs resulted in 30%, 68%, and 84% of the study’s cohort being categorized as obese, respectively [72]. Similarly, when the veterans were classified as having a WC > 102 cm [59], 69% met the obesity cutoff criteria. When using an SCI-specific cutoff of 94 cm [69], 77% of the veterans met the criteria [72].

Obesity has been used to indicate cardiometabolic risk after SCI. In a multicenter study at eight SCI rehabilitation centers in the Netherlands, Dorton et al. [73] identified 257 people with chronic traumatic SCI and compared BMI, WC, and waist-to-hip ratio to cardiovascular disease risk. The authors reported that WC, compared to BMI and waist-to-hip ratio, had the strongest correlation with—and the largest area under—the curve of the Framingham Risk Score 10-year cardiovascular disease risk [73]. Mercier et al. [74] reported that obesity (defined as a BMI ≥ 22 kg/m^2^) was prevalent (82%) and co-occurred with most other CMS risk factors in a retrospective cohort study in 103 adults with SCI. Likewise, in the Swedish Aging with SCI Study, Jörgensen et al. [75] revealed that 60% of the participants with SCI had a BMI > 22 kg/m^2^ associated with cardiometabolic risk. The authors also noted that 93% of the participants were considered obese/overweight using the SCI-adjusted BMI of 22 kg/m^2^ [75]. Using the gold standard 4-compartment modeling to measure body composition, Gater et al. [76] reported that 97% of people with motor complete SCI were obese. The authors demonstrated that a BMI of 27.3 ± 5.9 kg/m^2^, representing an “overweight” BMI category, corresponded to a total %BF of 42.4 ± 8.6% [76]. Of note, this latter value greatly exceeds the traditional %BF definition of obesity (men > 22% and women > 35%). Yoon et al. [77] examined the association of insulin resistance, low-grade systemic inflammation, and markers of subclinical atherosclerosis in people with SCI classified with metabolically healthy obesity (defined as an SCI-specific BMI > 22 kg/m^2^ with <3 metabolic abnormalities), metabolically unhealthy obesity, and metabolically healthy normal weight. The authors observed that despite similar metabolic and inflammatory statuses, people with both SCI and metabolically healthy obesity present with increased aortic stiffness but not carotid thickness. Yoon et al. [77] concluded that people with both SCI and metabolically healthy obesity demonstrate an intermediate subclinical atherosclerotic phenotype. Using dual X-ray absorptiometry (DXA) to quantify visceral fat in SCI participants, Cirnigliaro et al. [78] found that cardiometabolic risk was associated with central obesity. The authors reported that compared to SCI people below the cutoff threshold, SCI people with a visceral fat volume above the cutoff value of 1630 cm^3^ were 3.1-times more likely to have elevated serum triglycerides, 4.8-times more likely to have low serum HDL-C, 5.6-times more likely to have insulin resistance, 19.2-times more likely to have CMS, and 16.7-times more likely to have a 10-year Framingham Risk Score ≥ 10%.

Magnetic resonance imaging (MRI) has been utilized to quantify abdominal obesity after SCI, given the cardiometabolic risk associated with visceral fat. Early work by Gorgey and colleagues [68] using MRI to assess abdominal fat suggested that a ratio of visceral-to-subcutaneous fat > 0.4 increases cardiometabolic risk in individuals with SCI. In SCI, studies have since demonstrated that men, but not women, present with a visceral-to-subcutaneous fat ratio above 0.4 [5,79]. When disregarding sex differences, individuals with paraplegia and tetraplegia present with a ratio > 0.4 [80,81]. Farkas et al. [80] reported significant correlations among both MRI-assessed visceral fat and the visceral-to-subcutaneous fat ratio with triglycerides, HDL-C, and the TC:HDL-C ratio in paraplegia, but not tetraplegia [80]. Similarly, Gorgey et al. [82] observed several significant correlations between measures of lipid metabolism and abdominal obesity measured by MRI in SCI. The authors reported that HDL-C, TC:HDL-C ratio, and triglycerides correlated to upper and lower visceral and subcutaneous fat and the ratio of the two locations [82]. Interestingly, Rankin et al. [83] quantified the visceral fat around the liver using MRI in people with SCI and reported that it was positively related to total visceral fat, TNF-α, and several markers of cardiometabolic profile [83].

Over the last few years, studies have demonstrated obesity and cardiometabolic risk in acute SCI. Solinsky and colleagues [84] compared participants with acute SCI to age-, sex-, and BMI-matched controls from the National Health and Nutrition Examination Survey. The authors identified that 31.6% of participants with SCI had ≥3 cardiometabolic risks. This finding was significantly higher than the 22.3% identified in the matched controls [84]. Using data from the National SCI Statistical Center, Wen et al. [85] investigated the association between BMI and one-year mortality among people who survived the first 90 days after an SCI. The authors reported, based on BMI obtained during the initial rehabilitation, that the one-year mortality rates for people with SCI defined as overweight (25–29.9 kg/m^2^) and obese (≥30 kg/m^2^) were 3.1% and 3.5%, respectively [85]. Alternatively, the one-year mortality rates for underweight (<18.5 kg/m^2^) and normal weight (18.5–24.9 kg/m^2^) were 2.6% and 1.8%, respectively [85]. This study further detailed that those individuals with SCI with obesity had a higher hazard ratio of 1.51 for mortality risk than those with normal weight, citing the most frequent causes of death for SCI people with obesity were infective and parasitic diseases and respiratory diseases [85].

Studies have examined sex-based differences in cardiometabolic health after SCI. Gater et al. [76] reported that males with chronic motor complete SCI had significantly greater supine WC (M: 95 ± 12 vs. F: 86 ±13) and sitting sagittal (M: 32 ± 5 vs. F: 27 ± 7) and transverse (M: 36 ± 5 vs. F: 27 ± 7) abdominal diameters than females. Farkas et al. [5] and Gorgey et al. [82] demonstrated sex-based differences in MRI-assessed central obesity in adults with chronic motor complete SCI. Farkas et al. [5] reported that visceral fat was significantly greater in men, whereas subcutaneous fat was significantly greater in women with SCI. Interestingly, total trunk adipose tissue did not differ by sex [5]. The sex-specific accumulation of fat may account for the greater cardiovascular risk in men with SCI. Gater et al. [76], Farkas et al. [5], and Gorgey et al. [82] observed poorer cardiometabolic health in men compared to women with SCI [5]. This likely relates to the reduced testosterone levels in men with SCI compared to men without SCI [86,87]. Collectively, sex does matter with regards to obesity in people with SCI. More emphasis should be placed on sex differences in the causes, prevention, and management of obesity and its related complications in this unique population.

The influence of the level of SCI on obesity has remained relatively controversial until recently [80]. In a recent systematic review and meta-analysis, Raguindin et al. [88] pooled 40 studies, including 4872 people with chronic SCI (3991 men, 825 females, and 56 sex-unknown; 12.3 years median time since injury). The authors reported that despite a lower BMI in people with tetraplegia compared to paraplegia, those with tetraplegia had a 1.9% higher amount of total %BF, a 3.0 kg lower amount of lean mass, a 24 cm^2^ higher area of visceral fat, and a 1.05 L higher volume of visceral fat [88]. This study confirms that tetraplegia results in higher total and regional obesity than paraplegia.

Psychosocial and socioeconomic factors after SCI also influence obesity patterns. In a large epidemiological study, Graupensperger et al. [89] reported an age-adjusted odds ratio of 3.08 for being overweight/obese in 3136 people with SCI compared to 758,462 controls. The authors identified that people with SCI had increased odds of co-occurrence of overweight/obese and anxiety (odds ratio = 4.30) or depressive (odds ratio = 4.69) disorders compared to controls [89]. Within the SCI cohort, Graupensperger et al. [89] found that for people with SCI, those who were overweight/obese had greater odds of having anxiety (odds ratio = 2.54) or depressive (odds ratio = 2.70) disorders than non-overweight/obese individuals with SCI. Wen and associates [90] studied the role of neighborhood characteristics in the relation between race and obesity for people with SCI. The authors utilized data from the National SCI Statistical Center database linked with neighborhood data from the American Community Survey by census tract [90]. After controlling for demographic and injury-related characteristics, Wen et al. [90] showed that Hispanic people with SCI were 67.0% more likely to be obese (defined as a BMI ≥ 30.0 kg/m^2^) than non-Hispanic whites with SCI [90]. After accounting for the concentrated disadvantage index (the proportion of households in census tracts with a high level of concentrated disadvantage), the odds of obesity in Hispanics with SCI decreased by 51% [90]. Regardless of race and ethnicity, people from disadvantaged neighborhoods with SCI were 42.0% to 70.0% more likely to be obese than people from disadvantaged neighborhoods without SCI. In a similar analysis regarding race in people with SCI by Wen et al. [91], the authors assessed differences in BMI change over five years. In this population, the authors reported the greatest BMI increases in individuals that identified as Hispanics, followed by non-Hispanic Whites and non-Hispanic Blacks [91].

Obesity is a complex, multifactorial chronic disease that becomes even more problematic after an SCI. The factors contributing to and developing from neurogenic obesity remain a public health concern for the population with SCI. The cardiometabolic sequalae stemming from the vast accumulation of adipose tissue after the injury provides additional evidence to observations from the general population that obesity is a primary driver of adverse health outcomes.

## 4. Dyslipidemia after SCI

Dyslipidemia—including hypertriglyceridemia, hypercholesterolemia, and hypoalphalipoproteinemia—is a widely studied component risk factor for cardiometabolic risk and CMS after SCI (Table 4). Compared to controls without SCI from the National Health and Nutrition Examination Survey, Solinsky et al. [84] observed that people with acute SCI had significantly higher triglycerides and lower HDL-C. Specifically, low HDL-C was also observed in 54.2% of participants with SCI compared to only 15.4% of the controls [84]. In one of the most extensive epidemiological studies on cardiometabolic morbidities after SCI, Peterson et al. [12] compared 9081 adults with SCI to approximately 1.5 million adults without an SCI from longitudinal data in a nationwide insurance claims database from the US. The authors reported that people living with traumatic SCI, compared to controls, had a higher 5-year incidence (SCI: 25.5% vs. Controls: 16.9%, respectively) and 1.53 (53%) greater hazard for hypercholesterolemia [12]. DiPiro et al. [92] identified hypercholesterolemia in the US in 32.2% of the registrants from the South Carolina SCI Surveillance System Registry (*n* = 787). In the same registry, Cao et al. [93] assessed the changes in chronic health conditions over a four-year interval in people with longstanding SCI. They reported that the prevalence of hypercholesterolemia significantly increased from 32% to 44% [93]. The latter number is similar to that of the general population in South Carolina [94]; however, the increase over the four-year interval remains poorly understood.

Similar findings regarding dyslipidemia have also been reported internationally. Tallqvist et al. [95] surveyed the Finnish population with SCI and observed that 22% had hypercholesterolemia. This value was less than half that of the general population in Finland, where approximately 55% have hypercholesterolemia [96]. Conversely, Jörgensen et al. [75] showed that dyslipidemia was present in 76% of the respondents from the Swedish Aging with SCI Study, whereas 16% had pre-diagnosed dyslipidemia and 60% had hyperlipidemia [75] (values above historical norms for the country [97]). The authors further elucidated that the most common dyslipidemic profile for people with dyslipidemia was an elevated LDL-C [75]. In 269 people with SCI from Turkey, Koyuncu and colleagues [98] identified that TC, LDL-C, and triglycerides were 21%, 24%, and 31% higher than standard cutoffs in people with SCI. HDL-C was <40 mg/dL in 80% of the participants, while the TC:HDL-C ratio was ≥4.5 in 66% of the study sample, further supporting a dyslipidemic profile. Koyuncu et al. [98] noted HDL-C levels in motor complete SCI were significantly lower than those with motor incomplete SCI [98]. The TC:HDL-C ratio was significantly higher in people with SCI with a disease duration of ≤12 months than in the group with a longer disease duration [98].

Differences in lipid profiles have been observed by level of injury and sex for people with SCI. Sabour et al. [99], Jörgensen et al. [75], and Farkas et al. [5] found that men with SCI had significantly lower HDL-C levels than women with SCI [75]. In addition, Farkas et al. [5] observed that men with SCI had a higher TC:HDL-C ratio than women with SCI. Sullivan and colleagues [86] reported that in men with SCI, those with low free and total testosterone had significantly lower HDL-C levels without differences in fasting triglycerides or LCL-C than men with normal testosterone levels. Among men with SCI, Abilmona et al. [87] identified that in those with normal serum testosterone, serum triglycerides were 41% below that of men with low range serum testosterone levels, providing evidence that sex-based hormones may influence lipid profiles after SCI. Regarding the level of SCI, Wahl and Hirsch [44] noted in a systematic review that people with paraplegia had a greater occurrence of dyslipidemia than people with tetraplegia. La Fountaine et al. [100] reported that people with SCI below T5 presented with significantly higher serum triglycerides and higher very LDL-C concentrations than people with an SCI above T4 and the control group without SCI. The following year, La Fountaine et al. [101] identified that a lower triglyceride cutoff value was associated with dyslipidemia in people with SCI (115 mg/dL in SCI above T4 and 137 mg/dL in SCI below T5) than in people without SCI. Similarly, in another study, people with injuries below T6 had a higher rate of hypercholesterolemia than people with SCI above T7 [102]. These studies suggest that male gender and level of injury are unmodifiable cardiovascular risk factors (Figure 1), and standard cutoffs for hypertriglyceridemia may be inappropriate for SCI.

**Table 4:** Critical studies enumerating cardiometabolic risk after SCI over the last five years.

				Cardiometabolic Risk Factor		
Paper	Country	Sample Size (*n*)	SCI	Dyslipidemia	Hypertension	Dysglycemia/Insulin Resistance
Adriaansen et al., 2017 [103]	Netherlands	282	Chronic		21.50%	
Aidinoff et al., 2017 [102]	Israel	154	Chronic		T4-T6: 52% vs. >T4: 23.3%	
Cao et al., 2020 [93]	USA	501	Chronic	Hypercholesterolemia: 4-year increase 32–44%		Diabetes: 4-year increase 14–17%
DiPiro et al., 2018 [92]	USA	787	Chronic	Hypercholesterolemia: 32.3%	43.10%	Diabetes: 15.8%
Gater et al., 2019 [104]	USA	473	Mixed	Hypercholesterolemia: 69.7% Hypertriglyceridemia: 37.1%	55.10%	Diabetes: 49.7%
Gater et al., 2021 [76]	USA	72	Chronic	Hypercholesterolemia: 83% Hypertriglyceridemia: 33%	43%	Hyperglycemia: 32%
Jörgensen et al., 2019 [75]	Sweden	123	Chronic	Dyslipidemia: 76% Hyperlipidemia: 60%	33% diagnosed 55% undiagnosed	Diabetes: 16% Impaired Fasting Glucose: 27% Hyperglycemia: 15%
Koyuncu et al., 2017 [98]	Turkey	269	Mixed	High Total Cholesterol: 21% High LDL-C: 24% Hypertriglyceridemia: 31% Hypoalphalipoproteinemia: 80%		
Peterson et al., 2021 [12]	USA	9081	Unknown	Hypercholesterolemia: 5-year incidence, SCI: 25.5% vs. Controls: 16.9%, 1.53 greater hazard for SCI vs. controls	5-year incidence, SCI: 43.7% vs. Controls: 24.8%, 1.82 greater hazard for SCI vs. controls	
Solinsky et al., 2021 [84]	USA	95	Acute	Hypoalphalipoproteinemia: 52.4%		Hyperglycemia: 12.5% Insulin Resistance: 33.3%
Tallqvist et al., 2021 [95]	Finland	884	Chronic	Hypercholesterolemia: 22%	40%	
Ullah et al., 2018 [105]	Saudi Arabia	24	Acute		75%	Diabetes: 60%
Vriz et al., 2017 [106]	Italy	57	Chronic		11%	

Dyslipidemia after SCI contributes to the population’s increased cardiometabolic risk. An unfavorable lipid profile is typically a modifiable risk factor, dependent upon lifestyle modifications and/or pharmacological intervention. However, for people with SCI, the severity of dyslipidemia is reportedly more strongly related to time since the SCI than to diet [107], suggesting that time since injury may potentially be a non-modifiable factor of cardiometabolic risk.

## 5. Hypertension after SCI

Autonomic nervous system dysregulation leads to the interruption of normal cardiovascular homeostasis. This disruption increases the risk of hemodynamic instability, especially at higher injury levels. While paradoxical to SCI-induced neurogenic hypotension due to sympathetic dysfunction, hypertension remains a prevalent cardiometabolic risk factor after SCI (Table 4). Peterson et al. [12] reported that individuals living in the US with traumatic SCI had a greater 5-year incidence of hypertension (43.7% vs. 24.8%, respectively) and 1.82 (82%) greater hazard for hypertension compared to controls. In the Netherlands, Adriaansen et al. [103] identified that the prevalence of hypertension was 21.5% in 282 Dutch people with long-term SCI that included primarily men (74.1%). In comparison, the overall prevalence of hypertension in the general Dutch population is 21.4% in men and 14.9% in women [108]. In the US, DiPiro et al. [92] found a prevalence of hypertension in 43.1% of 787 adults with chronic SCI registered in the South Carolina SCI Surveillance System Registry (roughly 40% of the adults in the general population in South Carolina have hypertension [109]). Ullah et al. [105] studied hypertension in a small sample (*n* = 24) of elderly (72.3 years old) Saudi Arabians with spinal cord injuries/disorders. The authors observed that hypertension was the most common comorbidity, such that 75% of the study participants presented with the condition compared to 26.1% in the general Saudi Arabian population of all ages [110]. Tallqvist et al. [95] reported that among the Finnish SCI population, hypertension was found in almost 40% of the 884 participants surveyed, which was 3% below that of the general population [111]. Jörgensen et al. [75], in the Swedish Aging with SCI Study, demonstrated that 33% of the study’s cohort had a previous diagnosis of hypertension, and 55% presented with a hypertensive blood pressure ≥ 140/90 mmHg (27% of the general Swedish population are said to have hypertension [112]). The authors also reported that older chronological age, older age at injury, and shorter time since injury were significantly associated with higher systolic blood pressure [75].

Studies have investigated the influence of the level of injury on hypertension. Increased cardiovascular risk is especially marked in people with high levels of injuries [113] as these people experience repetitive and severe bouts of episodic hypertension (≤300 mmHg) during autonomic dysreflexia (AD; a transient hypertensive condition), which can occur over 40 times per day [114,115]. AD is a medical emergency that requires immediate treatment to remove the precipitating stimuli and, in severe situations, pharmacological stabilization of blood pressure [116]. Alternatively, while lower levels of injuries are less associated with AD, they are still associated with hypertensive risk. In Italy, Vriz et al. [106] reported that at a 7-year follow-up, nearly 11% of individuals with paraplegia demonstrated elevated blood pressure and were significantly heavier, with a tendency toward increased abdominal obesity after adjustment for age and systolic blood pressure. Adriaansen et al. [103] identified that the significant predictors of hypertension were injury levels below C8 (specifically, T1-T6 with an odds ratio of 6.4 and T7-L5 with an odds ratio of 10.1), a history of hypercholesterolemia (odds ratio = 4.8), longer time since injury (odds ratio = 1.1), and older age (odds ratio = 1.1). Moreover, in Dutch individuals, Adriaansen et al. [103] reported that the prevalence of hypertension and/or the use of antihypertensive medications was higher in men (T1-T6 lesion: 48%; and T7-L5 lesion: 57%) and women (T1-T6 lesion: 48%; T7-L5 lesion: 25%) with an SCI below C8 than men (31%) and women (18%) without an SCI [103]. In a retrospective observational comparative study, Aidinoff and colleagues [102] compared 154 Israelis with traumatic and non-traumatic SCI to Israeli and US general-population data adjusted for age, gender, and years of education. The authors reported that hypercholesterolemia (relative risk = 2.0) and older age at injury (relative risk = 1.06) significantly increased the hazard of hypertension. Hypertension was also significantly more prevalent at the T4-T6 injury level than those above T4 (52% vs. 23.3%, respectively) [102], suggesting that people with lower injury levels are at greater hypertensive risk.

At face value, it appears that people with lower rather than higher injury levels are at greater risk for hypertension. However, because people with injuries above T6 are likely to present with a low resting blood pressure, their elevated levels may not reach the thresholds for hypertension diagnosis as typically measured by a sphygmomanometer (Table 1). Consequently, these individuals may be overlooked for the diagnosis of hypertension and subsequently CMS as diagnostic thresholds may not be reached. Nevertheless, research has shown that SCI-induced autonomic nervous system dysregulation is associated with cardiovascular risk [117]. Therefore, hypertension in people with high injury levels is likely being underdiagnosed and misrepresented by current classifications defined for the population without SCI.

## 6. Dysglycemia and Insulin Resistance after SCI

Studies have reported on disorders of carbohydrate metabolism following SCI, including insulin resistance, type 2 diabetes mellitus, and pre-diabetes (Table 4). Li et al. [118] observed no differences in the concentration of measures of fasting glucose, insulin, and C-peptide among women with tetraplegia, paraplegia, and controls without SCI. However, the measures at minute 120 during an oral glucose tolerance test were higher in the former group compared to the other two groups [118]. Further, women with tetraplegia had a lower insulin sensitivity index compared to controls without SCI, even after adjusting for visceral fat and total body lean mass [118]. Peterson et al. [12] calculated a hazard ratio of 1.72 concerning type 2 diabetes mellitus in SCI and an incidence of 15.9% and 9.2% in people with and without SCI, respectively. DiPiro et al. [92] found a diabetes prevalence of 15.8% in 787 adults with chronic SCI from the South Carolina SCI Surveillance System Registry. Cao et al. [93] reported that among the registry participants, the prevalence of diabetes significantly increased from 14% at baseline to 17% during a 4-year interval follow-up [93]. In adults living in South Carolina without SCI, the latest data show the prevalence of diabetes is at 13.3%, a 1.2% increase from 2011 [119], suggesting that people with SCI living in South Carolina may be at greater risk for diabetes. Jörgensen et al. [75] revealed that in Swedish people with SCI, 16% had a history of diabetes, 27% had impaired fasting glucose, and fasting glucose levels were ≥126 mg/dL in 15% of the participants. These values generally exceed the limited data on carbohydrate metabolism in the general Swedish population [120]. Ullah et al. [105] reported that among a geriatric population of Saudi Arabian with SCI, diabetes mellitus was nearly 60% (versus 24% in the general Saudi Arabian population [121]). Chen et al. [122] reported that the prevalence of diabetes increased with age among 11,598 individuals living with SCI from the SCI Model Systems Database, mirroring the general population [123,124]. Additionally, Chen and colleagues [122] noted that the increased prevalence of diabetes among older individuals with SCI was consistent across all neurological groups, such that C1-C4 ABC injuries presented with the highest prevalence. These findings are supported by Wahl and Hirsch [44], who reported that people with tetraplegia were more likely to have diabetes than people with paraplegia [44].

Many techniques are used to assess carbohydrate profiles after SCI; however, not all assessment measures are equal. In men with SCI, Sullivan and colleagues [86] observed that those with low total and low free testosterone had significantly greater fasting glucose and insulin resistance without differences in percent hemoglobin A1C than those with normal testosterone levels. Solinsky et al. [84] identified insulin resistance in 12.5% of their cohort using elevated fasting plasma glucose as a criterion but in 33.3% when using Homeostatic Model Assessment 2 for Insulin Resistance (HOMA2-IR) criteria. Farkas and colleagues [125] recently examined the accord among indices of glucose metabolism against the gold standard measure of insulin sensitivity as assessed by the intravenous glucose tolerance test in 29 people with chronic motor complete SCI (79% men, 42.2 ± 11.4 years old, BMI 28.6 ± 6.4 kg/m^2^, C4 to T10). The authors demonstrated that the greatest agreement with insulin sensitivity was with the Quantitative Insulin-sensitivity Check Index (QUICKI), followed by Homeostatic Model Assessment for Insulin Resistance, HOMA2-IR, and the Matsuda Index. Despite being commonly used for evaluating disorders of carbohydrate metabolism, fasting plasma glucose and hemoglobin A1C had the poorest agreement with insulin sensitivity. Farkas et al. [125] hypothesized that QUICKI’s superior agreement stems from the log transformation of QUICKI values. The authors noted that for people with SCI, in the absence of QUICKI, fasting plasma glucose and hemoglobin A1C should be used in combination rather than in isolation to provide better diagnostic utility.

## 7. Cardiometabolic Syndrome after SCI

The 2018 Paralyzed Veterans of American Consortium for Spinal Cord Medicine Clinical Practice Guidelines on Identification and Management of Cardiometabolic Risk after Spinal Cord Injury (PVA Guidelines) [45] published recommendations for identifying and managing cardiometabolic risk and CMS for people with SCI (Table 5). These guidelines align with current recommendations for identifying and managing the cardiometabolic risk in people without SCI (Table 1); however, the PVA Guidelines incorporate the unique pathophysiology of SCI in their recommendations.

Globally, the prevalence of CMS is estimated to be about 25%, resulting in over one billion impacted people [127]. In the US, the prevalence of CMS was 34.7% in the general population from 2011 to 2016 [128]. The PVA Guidelines reported that the prevalence of CMS/disease ranges from 31% to 72% in the adult population with SCI. The sizable range in the prevalence of CMS in people with SCI results from the CMS definition, heterogeneity of the study participants, and study sample size. However, recent work from our laboratory has underscored the alarming prevalence of cardiometabolic risk in both veterans and civilians with SCI. In 473 veterans with SCI, Gater et al. [104] reported that 76.7% were obese when assessed by the SCI-adjusted BMI cutoff of 22 kg/m^2^ [62], and 55.1% had, or were undergoing treatment for, hypertension; nearly 50% currently had, or were previously diagnosed with, type 2 diabetes mellitus; 69.7% had, or were under treatment for, HDL-C < 40 mg/dL; and 57.5% had IDF-defined CMS [23]. The authors’ use of the IDF criteria reflects the Federation’s prioritization of the role of central obesity in the development of CMS, as visceral fat is marked by increased central girth [23]. In a recent study by Gater and associates [76], the authors examined IDF-defined CMS and risk factors in civilians with chronic motor complete SCI. The authors observed that 33% of the study participants had triglycerides ≥ 150 mg/dL, or were under treatment for hypertriglyceridemia; 83% had HDL-C below sex-specific thresholds, or were under treatment for hypoalphalipoproteinemia; 43% had or were under treatment for hypertension; and 32% had a fasting glucose ≥ 100 mg/dL, or were under treatment for hyperglycemia [76]. When using the population-specific BMI classification of obesity and 4-compartment model-derived %BF, 55.7% and 59.4% of participants had CMS, respectively [76]. Peterson et al. [12] compared the incidence of and adjusted hazards for cardiometabolic morbidities between people with and without SCI. The authors reported that adults living with traumatic SCI had a higher 5-year incidence of any cardiometabolic morbidities than adults without SCI (56.2% vs. 36.4%) [12]. Additionally, survival models demonstrated that adults with SCI had a greater hazard for any cardiometabolic morbidity (Hazard Ratio: 1.67) and all cardiometabolic disorders compared to controls [12]. Collectively, these data illustrate that the prevalence of CMS is potentially greater than recently estimated.

The diagnosis of CMS is contingent on the number of possible risk factors included in the definition and the definition itself. Mercier et al. [74] reported that age, but not the time since injury, could be a risk factor for CMS. The authors noted that age increased the odds of a CMS diagnosis by 1.05 per year, while time since injury was not related to the odds of CMS diagnosis [74]. Yahiro et al. [72] examined CMS in veterans with SCI according to the WHO [20], NHLBI/AHA [19], NCEP ATP III [17], and the IDF [23] criteria. The authors found that the prevalence of CMS was 17% based on NCEP ATP III criteria, 19% based on WHO, 31% based on IDF, and 53% based on NHLBI/AHA [72]. The highest prevalence was according to the NHLBI/AHA definition, and this was maintained throughout all neurological levels of injury and impairment scale categories [72]. Interestingly, Yahiro et al. [72] identified that the kappa-statistic between the definitions of CMS ranged from fair to moderate, with IDF and NCEP ATP III and NCEP ATP III and WHO having the best agreement. NCEP ATP III and NHLBI/AHA had the worst agreement [72]. When Yahiro and colleagues [72] examined cardiometabolic or cardiovascular risk according to the Edmonton Obesity Staging System [129,130], Cardiometabolic Disease Staging System [131,132], and Framingham Risk Score [133,134], the authors observed that 30%, 80%, and 68% of veterans with SCI were at risk, respectively. As is evident by these data, cardiometabolic risk after SCI is high, independent of the definition. There is currently little consensus on the optimal identification criteria for CMS outside the PVA Guidelines. Future research is needed to test the validity and reliability of these guidelines and whether they accurately predict cardiovascular disease risk and cardiovascular disease *per se* [24].

## 8. Cardiovascular Disease after SCI

In the US [135], heart disease is the leading cause of death. For people with SCI, cardiovascular disease is a principal concern [10], and it remains the second leading cause of death, following only respiratory disease [10]. Hypertension, hypercholesterinemia, and smoking are key risk factors for the development of heart disease. Still, several other medical conditions and lifestyle choices also place individuals at high risk, including diabetes, overweight/obesity, unhealthy diet, physical inactivity, and excessive alcohol use (Figure 1 and Figure 2). These cardiometabolic and modifiable lifestyle hazards are intensified after SCI, creating a physiological environment that favors cardiovascular risk. Many modifiable risk factors are highly prevalent following SCI, including a current or past history of smoking [136,137,138], poor diet [30], physical inactivity [139,140,141,142], high alcohol consumption [143,144,145], psychosocial and low socioeconomic status [146,147,148,149], and left ventricular structural changes [150,151,152] (Figure 1 and Figure 2). In a prediction model of cardiovascular risk across a median 5.7-year follow-up period, Barton et al. [153] reported that the Framingham Risk Score underestimated the number of cardiovascular disease events [153]. The model did not improve even after the authors added the neurological impairment scale, motor impairment, and level of injury to the model [153].

Myocardial infarction, cardiac arrest and dysrhythmias, coronary and peripheral artery disease, atherosclerosis, and stroke have received recent attention in the context of people with SCI. Cao et al. [93] did not report significant increases in the prevalence of heart attack, coronary artery disease, or stroke over a 4-year interval. Contrary to these findings, Peterson et al. [12] reported that individuals living with traumatic SCI vs. people without SCI had a greater 5-year incidence of cardiac dysrhythmias (34.8% vs. 16.5%, respectively), heart failure (16.9% vs. 4.9%, respectively), and peripheral and visceral atherosclerosis (24.7% vs. 8.0%, respectively). The authors also found that people living with SCI had greater hazards for cardiac dysrhythmias (hazard ratio = 2.24), heart failure (hazard ratio = 3.55), and peripheral and visceral atherosclerosis (hazard ratio = 3.38) relative to controls [12]. Aidinoff and colleagues [102] reported that coronary artery disease (SCI: 11.7% vs. Israeli general-population: 8.5%) and myocardial infarction (SCI: 6.7% vs. Israeli general-population: 6.6%) were generally elevated in people with SCI who survived until the end of the follow-up compared to Israeli general-population data. Interestingly, a BMI > 30 significantly increased the odds of developing coronary artery disease, while the presence of a partner significantly decreased the risk [102]. Wu et al. [154] studied the risk of stroke in 2806 people with SCI compared to 28,060 age-, sex-, and propensity score-matched control subjects. All participants were followed for four years unless they died or had a stroke. Wu et al. [154] identified the incidence rate of stroke was 5.96 per 1000 person-years in people with SCI compared to the controls. A stroke was significantly more likely to occur in individuals with SCI than in the control group (adjusted hazard ratio = 2.85) [154]. The authors also reported that the incidence of ischemic stroke was significantly higher than that of hemorrhagic stroke (incidence rate ratio = 3.42) [154]. Solinsky et al. [84] reported that elevated risks for myocardial infarction and stroke were associated with the TC:HDL-C ratio and triglyceride:HDL-C ratio in acute SCI.

Arterial stiffness after SCI has also gained attention. Wahl and Hirsch [44] performed a systematic review of 42 articles examining cardiovascular risk factors after traumatic SCI. The authors reported an increased risk for peripheral artery disease and arterial changes, including a reduction in lumen size, increased vessel wall tension, impaired reactive hyperemic response, a lack of reduced vascular resistance, and higher vascular stiffness. Miyatani et al. [155] studied the association between cardiovascular risk factors and abnormal arterial stiffness defined by a carotid-femoral pulse wave velocity ≥ 10 m/s in 19 people with chronic SCI. The authors reported that increased arterial stiffness was significantly associated with dichotomized age ≥ 52 years, systolic blood pressure ≥ 130 mmHg, heart rate ≥ 62 bpm, and paraplegia. In a prospective analysis, Vriz et al. [106] completed transthoracic echocardiography and one-point left common carotid artery color-Doppler on people with and without paraplegia. Vriz et al. [106] reported that despite a lower BMI and diastolic blood pressure compared to healthy controls, people with paraplegia had significantly higher carotid stiffness and lower arterial compliance after adjusting for age, sex, BMI, physical activity, and heart rate [106]. The authors observed that people with paraplegia had significantly lower tricuspid annular plane systolic excursion and right systolic myocardial contraction velocity, increased relative wall thickness, and impaired diastolic function [106]. Wahl and Hirsch [44] reported that people with paraplegia had a greater occurrence of peripheral artery disease compared to people with tetraplegia. Interestingly, Currie et al. [156] showed that increased arterial stiffness was correlated to both hypotensive events and the combined frequency of hypotensive with hypertensive events in individuals with injuries above T6. The authors hypothesized that blood pressure instability fluctuations might play a role in arterial stiffening following SCI [156].

In summary, despite clear data showing the increased prevalence of cardiovascular disease and its risk factors [157,158], little is known about the progression of cardiovascular disease *per se* in people with SCI. Despite what is known about cardiometabolic comorbidities after SCI, long-term follow-up studies have not been conducted to quantify the cardiovascular disease that develops from cardiometabolic risk or CMS. A reported gap in the literature is the absence of quality prospective trials evaluating the prevalence and impact of cardiometabolic disorders and corresponding cardiovascular disease complications and endpoints after SCI, especially compared to matched controls [159]. Such studies are imperative for understanding cardiovascular disease-specific changes, the risk they impose, and their true impact after SCI.

## 9. Diagnosis and Management of Cardiometabolic Risk and Syndrome after SCI

Diagnosis and management of cardiometabolic risk and CMS in people with SCI mirror the recommendations for people without SCI. Surveillance of cardiometabolic risk and CMS should commence during the acute phase of the SCI and continue thereafter on an annual basis for all adults. General guidelines for managing CMS include reducing component risks to under three factors. Other modifiable cardiometabolic risk factors should also be targeted to optimize cardiovascular health (Figure 3). In fact, in developed countries, at least one-third of all cardiovascular disease is attributed to five risk factors: smoking/tobacco use, alcohol use, hypertension, hypercholesterinemia, and obesity [14,160]. Suboptimal diet [161] and sedentary behavior/physical inactivity [8,9] are also among the leading modifiable risk factors for cardiovascular disease and all-cause mortality worldwide. For people with SCI, a sedentary lifestyle is either involuntarily or voluntarily adopted [139,140,141,142], and poor dietary patterns are often observed [29,30,32,34]. Although algorithms for mitigating CMS have not been designed using SCI-specific thresholds for each risk factor, current strategies can be generally used to guide risk reduction.

## 10. Diagnosis of Cardiometabolic Risk and Syndrome after SCI

For the population with SCI, cardiometabolic risk, including dyslipidemia, hypertension, dysglycemia, obesity, and CMS status, should be annually evaluated as they are likely more susceptible. In addition, other risk factors should be considered in the risk assessment, including smoking/tobacco use, physical inactivity, diet, alcohol consumption, socioeconomic status, and psychosocial health status (Figure 1 and Figure 2).

A full fasting blood lipid panel including TC (<200 mg/dL), HDL-C (>40 mg/dL for men and >50 mg/dL for women), LDL-C (<100 mg/dL), and triglycerides (<150 mg/dL) should be performed by primary care physicians or physiatrists with or without SCI board certification [17]. Elevated blood pressure (≥130/≥85 for systolic and diastolic pressures, respectively) readings should be confirmed on a separate patient visit to diagnose hypertension [22]. Resting blood pressure should be kept on file. Assessments should consider postural influences and blood pressure variability due to autonomic instability in diagnosing hypertension after SCI. When assessing blood pressure, a supine or seated position should be noted. Dysglycemia diagnosis should include the evaluation of type 2 diabetes mellitus and pre-diabetes based on fasting plasma glucose, the 2-h plasma glucose value after a 75-g oral glucose tolerance test, or hemoglobin A1C criteria (Table 6) [43]. If quantifying insulin resistance after SCI, it should be evaluated with the QUICKI given its accord with the intravenous glucose tolerance test [43,125] (Table 6). Current pharmacotherapy treatment should be considered a positive qualification for dyslipidemia, hypertension, and/or dysglycemia. Obesity should be evaluated using the SCI-adjusted BMI > 22 kg/m^2^, or preferentially, using total %BF. Adults with BMI > 22 kg/m^2^ should be considered at high risk for cardiometabolic risk/CMS. Using total %BF, obesity should be evaluated with the 3- or 4-compartment models. These obesity guidelines form the basis for the remaining information presented in this section.

BMI calculation for people with SCI requires precise and reliable height and body weight measurements. Froehlich-Grobe et al. [162,163] noted that self-reported height and body weight in people using a wheelchair [162] and with SCI [163] were prone to error, conceivably to a larger degree than in the general population. An anthropometer should be used to measure the height of a person with SCI in the supine position on a flat exam table. The anthropometer should be aligned parallel to the edge of the exam table to ensure it is not angled. To measure height, two flat boards should be placed at the cranial and caudal end of the body with the distance measured between the boards. Lower extremity contractures should be minimized by ranging the lower limbs. Measuring body weight in individuals with SCI can be challenging, requiring an expensive and wheelchair-accessible scale (including a scale that can accommodate powerchairs), independent or dependent transfers in and out of the wheelchair, a table/mat to transfer the individual, and computation (i.e., subtracting the weight of the wheelchair with and without the person in it) that leads to errors in obtaining accurate weight measurements. Height and weight can also be measured using the ruler function and total body scan feature on the DXA scanner, respectively. However, the latter has not been validated relative to total body weight measured on a scale in people with SCI.

The principal assumption of BMI is that body weight, when adjusted to height squared, is closely related to body fatness and associated morbidity and mortality [164,165]. However, that is not always the case. Some individuals who are overweight or obese by BMI standards do not carry excessive stores of fat (e.g., bodybuilders). In contrast, other individuals can have a BMI within the normal range but have a greater percentage of their body weight as fat (e.g., people with SCI) [166]. Several studies have demonstrated that people with SCI present with a total %BF significantly above the male and female cutoff values of 22% and 35%, respectively [29,64,76,78,82,167,168,169]. The question then arises regarding how to correctly evaluate, manage, and stratify cardiometabolic risk by body fatness in these individuals.

The few existing studies show little consensus on total %BF ranges and how they relate to cardiometabolic risk, cardiovascular disease, and mortality. To identify and monitor obesity after SCI, we propose a novel total %BF categorization system to use in conjunction with the cardiometabolic risk guidelines after SCI. This system presents new total %BF levels computed utilizing standard BMI categories and total %BF thresholds (men > 22% and women > 35%) via algebraic cross-multiplication [55,57,58]. Table 7 presents the categorization system according to the traditional BMI categories. We hypothesize that greater cardiometabolic risk and cardiovascular morbidity and mortality develop with increasing the category of fatness. Future research will need to examine the association between the categorization system and cardiometabolic and cardiovascular morbidity and mortality.

## 11. Lifestyle Modifications to Mitigate Cardiometabolic Risk and Syndrome

Lifestyle modifications after SCI focus on mitigating cardiometabolic risk by increasing energy expenditure through physical activity or exercise [49] and reducing energy intake via a heart-healthy dietary pattern [27,29,30,31,32,33,34]. Clinical trials investigating the impact of exercise *plus* dietary intervention on cardiometabolic risk have been limited to date [170,171]. Instead, the literature has primarily focused on each lifestyle intervention as a monotherapy. Of note, in a combined therapeutic approach, Bigford and colleagues [170] incorporated a calorie-restrictive Mediterranean-style diet (1200–2000 kcal/day), 3-times weekly circuit resistance exercise [172,173,174], and 16 educational sessions with a lifestyle coach [175] modeled after the Diabetes Prevention Program in three people with paraplegia for 6-months. The program resulted in a body mass reduction that exceeded the Diabetes Prevention Program criterion of 7%, demonstrating improvements in insulin resistance, HDL-C, and triglycerides [170].

## 12. Exercise to Reduce Cardiometabolic Risk and Syndrome after SCI

Exercise is central to developing and preserving physical capacity and cardiometabolic health. There is evidence that physical exercise is a successful countermeasure for preventing and treating cardiometabolic risk and CMS in people with [176] and without [8] SCI. Studies have shown that physical exercise improves risk factors of CMS, including obesity [168,177,178,179], insulin resistance/dysglycemia [180,181,182,183,184,185], dyslipidemia [177,179,184,185,186,187,188], and hypertension [168,177].

Authoritative guidelines for physical activity and exercise after SCI have addressed the benefit of activity countermeasures for cardiometabolic risk [45,189]. Participation in physical activity/exercise should include at least 30 min of moderate to vigorous-intensity aerobic exercise 3 times per week [189] or at least 150 min of moderate-intensity exercise per week [45]. Exercise sessions can be fulfilled by sessions of 30 to 60 min performed 3 to 5 days per week or by exercising for at least 3, 10-min sessions per day [45]. Physical exercise for people with SCI can be achieved using functional electrical stimulation (FES), neuromuscular electrical stimulation, volitional upper extremity exercise (i.e., arm-crank ergometry, hand cycling, wheelchair propulsion, circuit resistance training), and hybrid exercise approaches.

People with SCI perform less exercise and are more physically deconditioned than the population without SCI and other groups with disabilities [139,190,191]. Well-documented SCI-related barriers to exercise impede participation. These barriers include accessible exercise equipment, lack of access to and availability of adaptive fitness facilities, transportation, health care and fitness professionals lacking background knowledge to train people with SCI, and failure to provide an appropriate exercise routine based on the neurological injury level [192,193,194,195]. Additionally, high injury levels [196], injury completeness, and upper extremity overuse injuries [197,198,199] limit the benefits of exercise. While adaptable fitness centers provide access to specialized exercise equipment, most of the equipment engages upper-body musculature. Upper-limb musculature involves a two-fold to three-fold smaller muscle mass than the legs [200] and, consequently, has a limited capacity for expending energy. Thus, upper-body exercise seldom produces the energy expenditure needed to compensate for excessive energy intake [34,201] without the involvement of the lower limb musculature.

With paralysis, lower-body exercise can be achieved through FES-evoked leg cycle ergometry. FES cycling produces rhythmic contractions of paralyzed lower limb muscles [202]. This exercise modality allows individuals with little or no voluntary movement of the lower limb to pedal an indoor exercise bicycle on a stationary system. Computer-generated, low-level electrical pulses are transmitted via transcutaneous electrodes to the muscles of the lower limb. The electrical current evokes coordinated contractions and a pedaling motion that mimics voluntary exercise training on a bicycle. FES cycling has been used to stimulate strength [203,204,205], endurance [204,206], and muscle hypertrophy [202,207]. It also has the potential to improve energy expenditure [168]; increase cardiac stroke volume [208,209]; increase peak power output, peak oxygen consumption, and ventilatory rate [168,206,210,211]; reverse myocardial disuse atrophy [212]; increase HDL-C [213]; and improve body composition [60,168,213,214]. Despite the potential and availability of FES cycling, the system is not consistently implemented as a standard of care and component of the lifelong rehabilitation for eligible people with SCI responsive to transcutaneous neurostimulation.

## 13. Dietary Patterns to Reduce Cardiometabolic Risk and Syndrome after SCI

While physical exercise is one of the primary modalities for lowering cardiovascular risk, after SCI, some people cannot counteract excessive energy intake with only physical exercise. Change in diet/nutrition is a widely accepted recommendation for the treatment and prevention of CMS, and it targets the energy mismatch that leads to obesity [215]. Consequently, dietary modification represents a focus for cardiometabolic risk management and prevention in people with SCI.

Few studies have examined dietary interventions on cardiometabolic risk after SCI. Chen et al. [216] conducted a pilot study examining a weight loss program that included education on nutrition, exercise, and behavioral modifications in 16 people with chronic SCI who were overweight/obese. The authors utilized the time-calorie displacement diet theory. This diet emphasizes a large intake of high bulk, low energy-density foods, such as high-fiber grains, cereals, and fruits and vegetables [216]. It also emphasized a moderate intake of high energy-density foods, such as meats, cheeses, sugars, and dietary fats [216]. The dietary intervention resulted in weight loss and improvements in dietary intake, BMI, psychosocial and physical functioning, and several arthrometric measures, but not in a reduction of DXA-measured body fat [216]. In a randomized controlled trial, Allison et al. [217] studied the change in nutrient intake and inflammatory mediators following a 3-month anti-inflammatory diet in 20 people with SCI. Participants in the intervention group (*n* = 12) were instructed to eliminate foods associated with common food intolerances and those that may mediate inflammation (e.g., refined grains/sugars, hydrogenated fat), as well as increase their intakes of foods with established anti-inflammatory properties (e.g., fish, quinoa). Participants were also provided daily anti-inflammatory supplements in the form of Omega 3 soft gels, antioxidants, curcumin, and vegetable-based protein powder. Allison et al. [217] reported in the treatment group a significant reduction in dietary fat intake and an increase in protein intake, but no change in carbohydrate or energy intake. The treatment group showed a significant increase in some nutrients with anti-inflammatory properties (A, C, and E, and omega-3 fatty acids) and a decrease in some nutrients with proinflammatory properties (trans fatty acids, caffeine, and sodium). Regarding the intervention’s impact on inflammatory mediators, the treatment group showed significant reductions in interferon-y, interleukin-1β, and interleukin-6 [217].

The PVA Guidelines were the first comprehensive publication to provide evidence-based recommendations on heart-healthy eating for people with SCI. The guidelines recommend caloric assessment utilizing indirect calorimetry to determine energy expenditure and assess energy needs; to implement a heart-healthy dietary pattern focusing on fruits, vegetables, fish, poultry, whole grains, legumes, nuts, low-fat dairy, and non-tropical vegetable oils while limiting sweets, sugar-sweetened drinks, and red meats. The recommendations also limit dietary saturated fat to 5% to 6% of the total energy intake and limit daily sodium intake to ≤2400 mg for individuals with hypertension [218]. Overall, a reduced emphasis should be placed on restricting macronutrients in diets after SCI, but rather on providing a healthy *dietary pattern* as instructed by the Dietary Guidelines for Americans [219] and the PVA Guidelines [45].

The PVA Guidelines [45] provided dietary recommendations that were recently expanded and adapted into practical, consumer-based everyday recommendations [34]. Farkas et al. [34] further recommended adopting ≤ 2400 mg of sodium for all individuals with SCI, irrespective of hypertension status, given the high consumption of sodium-dense foods reported across the literature. The authors also stressed the importance of lean poultry, consisting of a moderate 3 to 4 oz portion, and the consumption of fish two times per week. Vegetables should be eaten between 3 to 4 servings per day. They should consist of the five vegetable subgroups (including dark green, red, and orange, legumes [beans/peas], starchy, and others). Fruits should favor whole fruits with 2–3 servings per day, and 100% fruit juices should be limited because of their added/high sugar content and inadequate fiber content. Emphasis should be placed on low-fat dairy in the form of cheese, yogurt, and milk in small amounts while limiting saturated fat intake below 5–6%. High-fat, sugar-based sweets and drinks should be replaced with fresh fruit and water, respectively. Flavored and unflavored carbonated water and zero-calorie liquid water enhancers can be used to provide variety and flavor to drinks. Red meat and sweets should be consumed only on special occasions such as special holidays, weddings, birthdays, vacations, etc. By following the above recommended healthy dietary pattern, people with SCI will naturally limit their intake of refined/simple carbohydrates, sodium, and saturated fat and increase the consumption of unsaturated fats and fiber. Such dietary patterns will also promote optimal ingestion of micronutrients.

We endorse the significance of annual dietary assessments (minimally) and nutrition education with a registered dietitian as part of the medical assessment and management for people with SCI. We recommend that in addition to assessing body composition as described above, registered dietitians should: (1) assess resting/basal metabolism through indirect calorimetry or, when unavailable, calculate resting/basal metabolism with the Nightingale and Gorgey [220], Chun et al. [221], or Buchholz et al. [222] SCI-specific prediction equation; (2) determine total daily energy expenditure utilizing the prediction equation by Farkas et al. [29] to estimate energy needs; (3) encourage adherence to the SCI heart-healthy dietary guidelines as a healthy lifestyle choice; (4) prescribe dietary supplements when specific vitamin and/or mineral deficiencies have been detected or to avoid them when appropriate nutrition/healthy dietary patterns are adequate; and (5) explore dietary irregularities specific to SCI (i.e., refraining from food groups that may affect bowel/bladder function). Periodic assessments with the health care team, including a registered dietitian, should be implemented to manage and prevent cardiometabolic risk after SCI and allow the individuals to take an active role in their overall health.

Lastly, vitamin and mineral supplements should not be considered a healthy diet sub-stitute. The emphasis must be on energy and nutritional requirements from a healthy dietary pattern high in plant-based and whole foods that do not strip the micronutrients through ex-treme processing. The interactions and combination of phytochemicals, fiber, and other nu-trients in food cannot be placed in a dietary capsule, even though taking a daily supplement may be easier than focusing on a healthy dietary pattern. While multivitamins have im-portance, they should not lead to complacency about following healthy lifestyle practices (e.g., regular exercise, healthy eating, not smoking, and monitoring blood pressure and lipid levels). Moreover, the absorption of vitamins and minerals tends to be greater from food than from dietary supplements.

## 14. Summary

Cardiometabolic risk and CMS are a grave global public health crisis for the population with SCI. The definition of a pandemic is “an epidemic occurring worldwide, or over a very wide area, crossing international boundaries and usually affecting a large number of people” [223]. Arguably, obesity and CMS are at pandemic levels for people with SCI. Body fat lies at the center of a public health crisis, representing a major cause in disease pathogenesis and a promising therapeutic target. Many people with SCI present with cardiometabolic risk factors, the most serious of which is obesity. Individually, each cardiometabolic risk factor conveys increased cardiovascular disease risk, but as an amalgamation, they pose an even greater hazard. Additionally, several other risk factors and SCI-specific non-modifiable risks can intensify cardiometabolic risk for people with SCI. Management measures should focus on annual risk factor surveillance and lifestyle modifications that incorporate physical exercise and a heart-healthy dietary pattern. A dual-management approach with physical exercise/activity and a heart-healthy dietary pattern offers a successful approach to improving cardiometabolic health after SCI.

## Figures and Tables

**Table 1:** The most recognized definitions for identifying and diagnosing cardiometabolic syndrome and its component risk factors.

		International Diabetes Federation [22,23]	National Cholesterol Education Project Adult Treatment Panel III [17]	National Heart, Lung, and Blood Institute/American Heart Association [18,19]	World Health Organization [20]	European Group for the Study of Insulin Resistance [21]
Required Criteria/Emphasis		Obesity Plus, any 2 of the following risk factors	None. Any 3 of the following risk factors	None. Any 3 of the following risk factors	Impaired fasting glucose, impaired glucose tolerance (prediabetes) or type 2 diabetes mellitus, and/or insulin resistance * Plus, any 2 of the following risk factors	Insulin resistance or fasting hyperinsulinemia (>75% percentile) Plus, any 2 of the following risk factors
Component Risk Factors						
Central Obesity		Waist circumference ≥ 102 cm in US men or ≥88 cm in US women †,††	Waist circumference ≥ 102 cm in men ‡ or ≥88 cm in women	Waist circumference ≥ 102 cm in men or ≥88 cm in women	Waist-to-hip ratio > 0.90 in men; Waist-to-hip ratio > 0.85 in women; and/or body mass index > 30 kg/m^2^	Waist circumference ≥ 94 cm in men or ≥80 cm in women
Dyslipidemia	Elevated triglycerides	Triglycerides ≥ 150 mg/dL, or on treatment for dyslipidemia	Triglycerides ≥ 150 mg/dL	Triglycerides ≥ 150 mg/dL, or on treatment for evaluated triglycerides	Triglycerides ≥ 150 mg/dL	Triglycerides > 150 mg/dL, HDL-C < 39 mg/dL in men and women, or on treatment for dyslipidemia
	Reduced HDL-C	HDL-C < 40 mg/dL in men or <50 mg/dL in women, or on treatment for dyslipidemia	HDL-C < 40 mg/dL in men or <50 mg/dL in women	HDL-C < 40 mg/dL in men or <50 mg/dL in women, or on treatment for reduced HDL-C	HDL-C < 35 mg/dL in men or <39 mg/dL in women	
Hypertension		Systolic blood pressure ≥ 130 or diastolic blood pressure ≥ 85 mmHg, or on treatment previously diagnosed hypertension	Systolic blood pressure ≥ 130, or diastolic blood pressure ≥ 85 mmHg	Systolic blood pressure ≥ 130, diastolic blood pressure ≥ 85 mmHg, or on treatment for or previously diagnosed with hypertension	Blood pressure ≥ 160/90 mmHg § Blood pressure ≥ 140/90 mmHg §	≥140/90 mmHg, or on treatment for hypertension
Dysglycemia		Fasting plasma glucose ≥ 100 mg/dL, or previously diagnosed type 2 diabetes mellitus	Fasting plasma glucose ≥ 100 mg/dL **/≥ 110 mg/dL **	Fasting plasma glucose ≥ 100 mg/dL, or on treatment elevated glucose	Impaired fasting glucose, impaired glucose tolerance (prediabetes), or type 2 diabetes mellitus	Fasting glucose ≥ 110 mg/dL (but not diabetes, <126 mg/dL)
Insulin Resistance		None.	None.	None.	Insulin resistance *	Insulin resistance or fasting hyperinsulinemia (>75% percentile)
Other		None.	None.	None.	Microalbuminuria: urinary albumin excretion rate ≥ 20 µg/min, or albumin:creatinine ratio ≥ 20 mg/g	None.

* Insulin sensitivity measured under hyperinsulinemic-euglycemic conditions; glucose uptake below the lowest quartile for the population under investigation. ** The 2001 definition identified elevated fasting plasma glucose ≥ 110 mg/dL. In 2004 this was revised to ≥100 mg/dL per the American Diabetes Association’s updated definition of impaired fasting glucose [18,24,25]. † If body mass index is >30 kg/m^2^, central obesity can be assumed, and waist circumference does not need to be measured. †† Europid/Sub-Saharan African/Eastern Mediterranean/Middle East populations ≥ 94 cm in men and ≥80 cm in women; South Asians/South & Central Americas population ≥ 90 cm in men and ≥80 cm in women; Chinese population ≥ 90 cm in men and ≥80 cm in women; Japanese population ≥ 90 cm in men and ≥80 cm in women. ‡ Some men can develop multiple cardiometabolic risk factors when the waist circumference is only marginally increased (e.g., 94 to 102 cm). Such individuals may have a strong genetic contribution to insulin resistance. They should benefit from changes in lifestyle habits, similar to men with categorical increases in waist circumference. § A blood pressure ≥ 160/90 mmHg was initially proposed by World Health Organization (WHO) [20] in 1998. Since then, many alternative thresholds have been proposed, including the European Group for the Study of Insulin Resistance (EGIR) [21], which defines hypertension as a blood pressure ≥ 140/90 mmHg. The WHO has since adopted the EGIR definition of hypertension [26].

**Table 2:** Body mass index and waist circumference with standard category thresholds and ranges.

Body Mass Index (kg/m^2^)		Waist Circumference (cm)		
Classification	Threshold/Range	Classification	Gender	Threshold
Underweight	<18.5	Obese	Men	>102
Normal	18.5–24.9			
Pre-Obesity/Overweight *	25.0–29.9			
Obese	≥30		Women	>88
Obese I	30.0–34.9			
Obese II	35.0–39.9			
Obese III	≥40			

* Pre-obesity is used by the World Health Organization, while the Centers use overweight for Disease Control and Prevention.

**Table 3:** Population-specific Body Mass Index (BMI) and Waist Circumference (WC) Thresholds in Spinal Cord Injury.

Author (Year)	BMI Cutoff (kg/m^2^)	WC Cutoff (cm)	Nationality	Sample Size (*n*)	Age (y)	Sex (% Male)	ISNCSCI *	Injury Duration (y)	Method of Calculation
Ayas et al. [63] (2001)	>25.3	N/A	American	197	51 ± 15	NP	T, P/C, I	18 ± 13	Sample median
Inayama et al. [66] (2014)	>22.5	>81.3	Japanese	74	46 ± 14	100	T, P/C, I	15 ± 10	Non-LR
Laughton et al. [62] (2009)	>22.1	N/A	Canadian	77	44 ± 12	82	T, P/C, I	15 ± 11	Piecewise LR, ROC
Shin et al. [65] (2022)	>22.8	N/A	Korean	157	49 ± 12	70	T, P/C, I	12 ± 8	ROC
Sumrell et al. [70] (2018)	N/A	>86.5	American	22	36 ± 10	100	T, P/C, I	8 ± 8	LR
Ravensbergen et al. [69] (2014)	N/A	>94.0	Canadian	27	40 ± 11	70	T, P/C, I	14 ± 10	ROC
Yun et al. [64] (2019)	>20.2	>81.3	Korean	52	42 ± 11	100	T, P/C, I	13 ± 8	ROC, Youden index
Pooled Data									
BMI (kg/m^2^)	>23.3	N/A	Multiple	557	50 ± 13	84	T, P/C, I	15 ± 11	Pooling data *
WC (cm)	N/A	>83.9	Multiple	175	43 ± 12	95	T, P/C, I	13 ± 9	Pooling data *

C, Complete; I, Incomplete; ISNCSCI, International Standards for Neurological Classification of SCI; LR, Linear regression; N/A, not applicable; NP, Not provided; P, Paraplegia; ROC, receiver-operator characteristic curve; T, Tetraplegia. * Pooled values calculated according to Farkas et al. [30].

**Table 5:** Guideline Definition for Cardiometabolic Syndrome for Adults with Spinal Cord Injury from the Paralyzed Veterans of American Consortium for Spinal Cord Medicine Clinical Practice Guidelines on Identification and Management of Cardiometabolic Risk after Spinal Cord Injury [45].

Any 3 of the Following Component Risk Factors to Diagnosis Cardiometabolic Syndrome after SCI	
Obesity *	Total percent body fat (%BF) as determined by 3- (i.e., dual X-ray absorptiometry) or 4-compartment models [56,126]. Classify adult SCI men with >22%BF and adult SCI women with >35%BF as obese
	- Or -
	Body mass index (BMI) > 22 kg/m^2^ [62] is the SCI-specific cutoff point for obesity
Elevated triglycerides	Triglycerides ≥ 150 mg/dL
Reduced HDL-C	HDL-C < 40 mg/dL in men or <50 mg/dL in women
Hypertension	Systolic blood pressure ≥ 130 mmHg, diastolic blood pressure 85, or use of medication for hypertension
Dysglycemia	Fasting glucose ≥ 100 mg/dL or use of medication for hyperglycemia

* Proxy markers and SCI-specific definitions of obesity are used to report obesity in adults with SCI because waist circumference cutoffs (≥102 cm in men or ≥88 cm in women) are not validated in this population.

**Table 6:** Criteria for the diagnosing of Dysglycemia and Insulin Resistance.

Criterion	Normal	Pre-Diabetes	Diabetes
Fasting Plasma Glucose (mg/dL)	<100	100–125	≥126
Oral Glucose Tolerance Test (mg/dL) *	<140	140–199	≥200
Hemoglobin A1C (%)	<5.7	5.7–6.4	≥6.5
	Normal	Insulin Resistance	
Insulin Resistance **	>0.339	≤0.339	

* 2 h, 75-g glucose load. ** Defined and calculated using the Quantitative Insulin-sensitivity Check Index.

**Table 7:** Proposed total percent body fat (%BF) threshold and ranges as they relate to standard body mass index categories.

Body Mass Index (kg/m^2^)		Proposed %BF Cutoffs/Ranges to Report Obesity *		
Category [57,58]	Threshold/Range	Category	Men	Women
Underweight	<18.5	Irregular	<13.6	<21.6
Normal	18.5–24.9	Healthy	13.6–18.26	21.6–29.1
Pre-Obesity/Overweight **	25.0–29.9	Pre-Obesity	18.3–22	29.2–34.9
Obese ^	≥30	Obese [55,56]	>22	>35
Obese I	30.0–34.9	Obese I	22–25.6	35–40.7
Obese II	35.0–39.9	Obese II	25.7–29.3	40.8–46.6
Obese III	≥40	Obese III	>29.3	>46.6

* Calculated using algebraic cross-multiplication. ** Pre-obesity is used by the World Health Organization, while the Centers use overweight for Disease Control and Prevention. ^ The Paralyzed Veterans of American Consortium for Spinal Cord Medicine Clinical Practice Guidelines on Identification and Management of Cardiometabolic Risk after Spinal Cord Injury [45] promote the use of an SCI-specific BMI-cutoff of >22 kg/m^2^ to define obesity.

## References

[1] Castro M.J., Apple D.F., Hillegass E.A., Dudley G.A. (1999). Influence of complete spinal cord injury on skeletal muscle cross-sectional area within the first 6 months of injury. Eur. J. Appl. Physiol. Occup. Physiol..

[2] Grimby G., Broberg C., Krotkiewska I., Krotkiewski M. (1976). Muscle fiber composition in patients with traumatic cord lesion. Scand. J. Rehabil. Med..

[3] Gorgey A., Dudley G.A. (2006). Skeletal muscle atrophy and increased intramuscular fat after incomplete spinal cord injury. Spinal Cord.

[4] Zleik N., Weaver F., Harmon R.L., Le B., Radhakrishnan R., Jirau-Rosaly W.D., Craven B.C., Raiford M., Hill J.N., Etingen B. (2018). Prevention and management of osteoporosis and osteoporotic fractures in persons with a spinal cord injury or disorder: A systematic scoping review. J. Spinal Cord Med..

[5] Farkas G.J., Gorgey A.S., Dolbow D.R., Berg A.S., Gater D.R. (2018). Sex dimorphism in the distribution of adipose tissue and its influence on proinflammatory adipokines and cardiometabolic profiles in motor complete spinal cord injury. J. Spinal Cord Med..

[6] Gorgey A.S., Ennasr A.N., Farkas G.J., Gater D.R. (2021). Anthropometric Prediction of Visceral Adiposity in Persons with Spinal Cord Injury. Top. Spinal Cord Inj. Rehabil..

[7] Groah S.L., Nash M.S., Ward E.A., Libin A., Mendez A.J., Burns P., Elrod M., Hamm L.F. (2011). Cardiometabolic Risk in Community-Dwelling Persons with Chronic Spinal Cord Injury. J. Cardiopulm. Rehabil. Prev..

[8] Booth F.W., Roberts C.K., Laye M.J. (2012). Lack of Exercise Is a Major Cause of Chronic Diseases. Compr. Physiol..

[9] Lavie C.J., Ozemek C., Carbone S., Katzmarzyk P.T., Blair S.N. (2019). Sedentary Behavior, Exercise, and Cardiovascular Health. Circ. Res..

[10] DeVivo M.J., Chen Y., Wen H. (2021). Cause of Death Trends among Persons with Spinal Cord Injury in the United States: 1960–2017. Arch. Phys. Med. Rehabil..

[11] Garshick E., Kelley A., Cohen S.A., Garrison A., Tun C.G., Gagnon D., Brown R. (2005). A prospective assessment of mortality in chronic spinal cord injury. Spinal Cord.

[12] Peterson M.D., Berri M., Lin P., Kamdar N., Rodriguez G., Mahmoudi E., Tate D. (2021). Cardiovascular and metabolic morbidity following spinal cord injury. Spine J..

[13] Després J.-P., Lemieux I. (2006). Abdominal obesity and metabolic syndrome. Nature.

[14] World Health Organization (2009). Risk Factors.

[15] Ash-Bernal R., Peterson L. (2006). The Cardiometabolic Syndrome and Cardiovascular Disease. J. CardioMetabolic Syndr..

[16] Grundy S.M., Cleeman J.I., Merz C.N.B., Brewer H.B., Clark L.T., Hunninghake D.B., Pasternak R.C., Smith S.C., Stone N. (2004). Implications of Recent Clinical Trials for the National Cholesterol Education Program Adult Treatment Panel III Guidelines. J. Am. Coll. Cardiol..

[17] National Cholesterol Education Program (NCEP) Expert Panel on Detection, Evaluation, and Treatment of High Blood Cholesterol in Adults (Adult Treatment Panel III) (2002). Third Report of the National Cholesterol Education Program (NCEP) Expert Panel on Detection, Evaluation, and Treatment of High Blood Cholesterol in Adults (Adult Treatment Panel III) final report. Circulation.

[18] Grundy S.M., Brewer H.B., Cleeman J.I., Smith S.C., Lenfant C. (2004). Definition of Metabolic Syndrome: Report of the National Heart, Lung, and Blood Institute/American Heart Association conference on scientific issues related to definition. Circulation.

[19] Grundy S.M., Cleeman J.I., Daniels S.R., Donato K.A., Eckel R.H., Franklin B.A., Gordon D.J., Krauss R.M., Savage P.J., Smith S.C. (2005). Diagnosis and management of the metabolic syndrome: An American Heart Association/National Heart, Lung, and Blood Institute scientific statement. Circulation.

[20] Alberti K.G., Zimmet P.Z. (1998). Definition, diagnosis and classification of diabetes mellitus and its complications. Part 1: Diagnosis and classification of diabetes mellitus provisional report of a WHO consultation. Diabet. Med..

[21] Balkau B., Charles M.A. (1999). Comment on the provisional report from the WHO consultation. Diabet. Med..

[22] Holt R.I.G. (2005). News and Views. Diabet. Obes. Metab..

[23] Alberti K.G.M.M., Zimmet P., Shaw J. (2006). Metabolic syndrome—A new world-wide definition. A Consensus Statement from the International Diabetes Federation. Diabet. Med..

[24] (2004). American Diabetes Association Standards of Medical Care in Diabetes. Diabet. Care.

[25] Nesto R.W. (2003). The relation of insulin resistance syndromes to risk of cardiovascular disease. Rev. Cardiovasc. Med..

[26] World Health Organization (2013). A Global Brief on Hypertension: Silent Killer, Global Public Health Crisis: World Health Day 2013.

[27] Farkas G.J., Gater D.R. (2019). Energy Expenditure and Nutrition in Neurogenic Obesity following Spinal Cord Injury. J. Phys. Med. Rehabil..

[28] Farkas G.J., Gater D.R. (2017). Neurogenic obesity and systemic inflammation following spinal cord injury: A review. J. Spinal Cord Med..

[29] Farkas G.J., Gorgey A.S., Dolbow D.R., Berg A.S., Gater D.R. (2019). Caloric intake relative to total daily energy expenditure using a spinal cord injury-specific correction factor: An analysis by level of injury. Am. J. Phys. Med. Rehabil..

[30] Farkas G.J., Pitot M.A., Berg A.S., Gater D.R. (2018). Nutritional status in chronic spinal cord injury: A systematic review and meta-analysis. Spinal Cord.

[31] Farkas G.J., Pitot M.A., Gater D.R. (2019). A systematic review of the accuracy of estimated and measured resting metabolic rate in chronic spinal cord injury. Int. J. Sport Nutr. Exerc. Metab..

[32] Farkas G.J., Sneij A., Gater D.R. (2021). Dietetics After Spinal Cord Injury: Current Evidence and Future Perspectives. Top. Spinal Cord Inj. Rehabil..

[33] Farkas G.J., Sneij A., Gater D.R. (2021). Energy Expenditure Following Spinal Cord Injury: A Delicate Balance. Top. Spinal Cord Inj. Rehabil..

[34] Farkas G.J., Sneij A., McMillan D.W., Tiozzo E., Nash M.S., Gater D.R. (2021). Energy expenditure and nutrient intake after spinal cord injury: A comprehensive review and practical recommendations. Br. J. Nutr..

[35] Gater D.R., Farkas G.J., Tiozzo E. (2021). Pathophysiology of Neurogenic Obesity After Spinal Cord Injury. Top. Spinal Cord Inj. Rehabil..

[36] Sakers A., De Siqueira M.K., Seale P., Villanueva C.J. (2022). Adipose-tissue plasticity in health and disease. Cell.

[37] Jensen M.D., Haymond M.W., Rizza R.A., Cryer P.E., Miles J.M. (1989). Influence of body fat distribution on free fatty acid metabolism in obesity. J. Clin. Investig..

[38] Abate N., Chandalia M., Snell P.G., Grundy S.M. (2004). Adipose Tissue Metabolites and Insulin Resistance in Nondiabetic Asian Indian Men. J. Clin. Endocrinol. Metab..

[39] Perseghin G., Ghosh S., Gerow K., Shulman G.I. (1997). Metabolic Defects in Lean Nondiabetic Offspring of NIDDM Parents: A Cross-Sectional Study. Diabetes.

[40] Gancheva S., Jelenik T., Álvarez-Hernández E., Roden M. (2018). Interorgan Metabolic Crosstalk in Human Insulin Resistance. Physiol. Rev..

[41] Van Der Kolk B.W., Goossens G., Jocken J.W., Blaak E.E. (2016). Altered skeletal muscle fatty acid handling is associated with the degree of insulin resistance in overweight and obese humans. Diabetologia.

[42] Shin S.W., Lee S.J. (2014). Ectopic Fat in Insulin Resistance, Dyslipidemia, and Cardiometabolic Disease. N. Engl. J. Med..

[43] Gordon P.S., Farkas G.J., Gater D.R. (2021). Neurogenic Obesity-Induced Insulin Resistance and Type 2 Diabetes Mellitus in Chronic Spinal Cord Injury. Top. Spinal Cord Inj. Rehabil..

[44] Wahl U., Hirsch T. (2021). A systematic review of cardiovascular risk factors in patients with traumatic spinal cord injury. Vasa.

[45] Nash M.S., Groah S.L., Gater D.R., Dyson-Hudson T.A., Lieberman J.A., Myers J., Sabharwal S., Taylor A.J. (2019). Identification and Management of Cardiometabolic Risk after Spinal Cord Injury. J. Spinal Cord Med..

[46] Hu F.B., Meigs J.B., Li T.Y., Rifai N., Manson J.E. (2004). Inflammatory Markers and Risk of Developing Type 2 Diabetes in Women. Diabetes.

[47] Hanley A.J., Festa A., D’Agostino R.B., Wagenknecht L.E., Savage P.J., Tracy R.P., Saad M.F., Haffner S.M. (2004). Metabolic and Inflammation Variable Clusters and Prediction of Type 2 Diabetes. Diabetes.

[48] Kotsis V., Stabouli S., Papakatsika S., Rizos Z., Parati G. (2010). Mechanisms of obesity-induced hypertension. Hypertens. Res..

[49] Nash M.S., Farkas G.J., Tiozzo E., Gater D.R. (2021). Exercise to mitigate cardiometabolic disorders after spinal cord injury. Curr. Opin. Pharmacol..

[50] Hoene M., Weigert C. (2007). The role of interleukin-6 in insulin resistance, body fat distribution and energy balance. Obes. Rev..

[51] Larsen C.M., Faulenbach M., Vaag A., Vølund A., Ehses J.A., Seifert B., Mandrup-Poulsen T., Donath M.Y. (2007). Interleukin-1–Receptor Antagonist in Type 2 Diabetes Mellitus. N. Engl. J. Med..

[52] Catrysse L., van Loo G. (2017). Inflammation and the Metabolic Syndrome: The Tissue-Specific Functions of NF-κB. Trends Cell Biol..

[53] Dunmore S.J., Brown J.E.P. (2012). The role of adipokines in β-cell failure of type 2 diabetes. J. Endocrinol..

[54] Oda E. (2008). The Metabolic Syndrome as a Concept of Adipose Tissue Disease. Hypertens. Res..

[55] Heyward V.H., Wagner D.R. (2004). Applied Body Composition Assessment.

[56] Heyward V.H. (2001). ASEP methods recommendation: Body composition assessment. J. Exerc. Physiol. Online.

[57] World Health Organization Body Mass Index-BMI. https://www.euro.who.int/en/health-topics/disease-prevention/nutrition/a-healthy-lifestyle/body-mass-index-bmi.

[58] Centers for Disease Control and Prevention Defining Adult Overweight and Obesity. https://www.cdc.gov/obesity/adult/defining.html.

[59] Klein S., Allison D., Heymsfield S.B., Kelley D.E., Leibel R.L., Nonas C., Kahn R. (2007). Waist Circumference and Cardiometabolic Risk: A Consensus Statement from Shaping America’s Health: Association for Weight Management and Obesity Prevention; NAASO, The Obesity Society; the American Society for Nutrition; and the American Diabetes Association. Obesity.

[60] Gater D.R., Farkas G.J. (2016). Alterations in Body Composition After SCI and the Mitigating Role of Exercise. Physiol. Exerc. Spinal Cord Inj..

[61] Silveira S., Ledoux T.A., Robinson-Whelen S., Stough R., Nosek M.A. (2017). Methods for classifying obesity in spinal cord injury: A review. Spinal Cord.

[62] Laughton G.E., Buchholz A.C., Ginis K.A.M., Goy R.E., The SHAPE SCI Research Group (2009). Lowering body mass index cutoffs better identifies obese persons with spinal cord injury. Spinal Cord.

[63] Ayas N.T., Epstein L.J., Lieberman S.L., Tun C.G., Larkin E.K., Brown R., Garshick E. (2001). Predictors Of Loud Snoring In Persons with Spinal Cord Injury. J. Spinal Cord Med..

[64] Yun J.-H., Chun S.-M., Kim J.-C., Shin H.-I. (2018). Obesity cutoff values in Korean men with motor complete spinal cord injury: Body mass index and waist circumference. Spinal Cord.

[65] Shin J.W., Kim T., Lee B.-S., Kim O. (2022). Factors Affecting Metabolic Syndrome in Individuals with Chronic Spinal Cord Injury. Ann. Rehabil. Med..

[66] Inayama T., Higuchi Y., Tsunoda N., Uchiyama H., Sakuma H. (2014). Associations between abdominal visceral fat and surrogate measures of obesity in Japanese men with spinal cord injury. Spinal Cord.

[67] Gorgey A.S., Mather K.J., Gater D.R. (2011). Central adiposity associations to carbohydrate and lipid metabolism in individuals with complete motor spinal cord injury. Metabolism.

[68] Gorgey A.S., Mather K.J., Poarch H.J., Gater D.R. (2011). Influence of motor complete spinal cord injury on visceral and subcutaneous adipose tissue measured by multi-axial magnetic resonance imaging. J. Spinal Cord Med..

[69] Ravensbergen H.J.C., Lear S.A., Claydon V.E. (2014). Waist Circumference Is the Best Index for Obesity-Related Cardiovascular Disease Risk in Individuals with Spinal Cord Injury. J. Neurotrauma.

[70] Sumrell R.M., Nightingale T., McCauley L.S., Gorgey A.S. (2018). Anthropometric cutoffs and associations with visceral adiposity and metabolic biomarkers after spinal cord injury. PLoS ONE.

[71] Gill S., Sumrell R.M., Sima A., Cifu D.X., Gorgey A.S. (2020). Waist circumference cutoff identifying risks of obesity, metabolic syndrome, and cardiovascular disease in men with spinal cord injury. PLoS ONE.

[72] Yahiro A.M., Wingo B.C., Kunwor S., Parton J., Ellis A.C. (2019). Classification of obesity, cardiometabolic risk, and metabolic syndrome in adults with spinal cord injury. J. Spinal Cord Med..

[73] Dorton M.C., Lucci V.-E.M., de Groot S., Loughin T.M., Cragg J.J., Kramer J.K., Post M.W.M., Claydon V.E. (2020). Evaluation of cardiovascular disease risk in individuals with chronic spinal cord injury. Spinal Cord.

[74] Mercier H.W., Solinsky R., Taylor J.A. (2022). Relationship of cardiometabolic disease risk factors with age and spinal cord injury duration. J. Spinal Cord Med..

[75] Jörgensen S., Hill M., Lexell J. (2019). Cardiovascular Risk Factors among Older Adults with Long-Term Spinal Cord Injury. PM&R.

[76] Gater D.R., Farkas G.J., Dolbow D.R., Berg A.S., Gorgey A.S. (2021). Body composition and metabolic assessment after aotor complete spinal cord injury: Development of a clinically relevant equation to estimate body fat. Top. Spinal Cord Inj. Rehabil..

[77] Yoon E.S., Heffernan K.S., Jae S.Y., Kim H.J., Bunsawat K., Fernhall B. (2018). Metabolically healthy obesity and subclinical atherosclerosis in persons with spinal cord injury. J. Rehabil. Med..

[78] Cirnigliaro C.M., La Fountaine M.F., Hobson J.C., Kirshblum S.C., Dengel D.R., Spungen A.M., Bauman W.A. (2021). Predicting cardiometabolic risk from visceral abdominal adiposity in persons with chronic spinal cord injury. J. Clin. Densitom. Off. J. Int. Soc. Clin. Densitom..

[79] Farooq A., Knez W.L., Knez K., Al-Noaimi A., Grantham J., Mohamed-Ali V. (2013). Gender Differences in Fat Distribution and Inflammatory Markers among Arabs. Mediat. Inflamm..

[80] Farkas G.J., Gorgey A.S., Dolbow D.R., Berg A.S., Gater D.R. (2017). The influence of level of spinal cord injury on adipose tissue and its relationship to inflammatory adipokines and cardiometabolic profiles. J. Spinal Cord Med..

[81] Gorgey A.S., Gater D.R. (2011). A Preliminary Report on the Effects of the Level of Spinal Cord Injury on the Association Between Central Adiposity and Metabolic Profile. PM&R.

[82] Gorgey A.S., Farkas G.J., Dolbow D.R., Khalil R.E., Gater D.R. (2017). Gender Dimorphism in Central Adiposity May Explain Metabolic Dysfunction After Spinal Cord Injury. PM&R.

[83] Rankin K.C., O’Brien L.C., Segal L., Khan M.R., Gorgey A.S. (2017). Liver Adiposity and Metabolic Profile in Individuals with Chronic Spinal Cord Injury. BioMed Res. Int..

[84] Solinsky R., Betancourt L., Schmidt-Read M., Kupfer M., Owens M., Schwab J.M., Dusseau N.B., Szlachcic Y., Sutherland L., Taylor J.A. (2021). Acute Spinal Cord Injury Is Associated with Prevalent Cardiometabolic Risk Factors. Arch. Phys. Med. Rehabil..

[85] Wen H., DeVivo M.J., Mehta T., Baidwan N.K., Chen Y. (2019). The impact of body mass index on one-year mortality after spinal cord injury. J. Spinal Cord Med..

[86] Sullivan S.D., Nash M.S., Tefara E., Tinsley E., Groah S. (2017). Relationship Between Gonadal Function and Cardiometabolic Risk in Young Men with Chronic Spinal Cord Injury. PM&R.

[87] Abilmona S.M., Sumrell R.M., Gill R.S., Adler R.A., Gorgey A.S. (2018). Serum testosterone levels may influence body composition and cardiometabolic health in men with spinal cord injury. Spinal Cord.

[88] Raguindin P.F., Bertolo A., Zeh R.M., Fränkl G., Itodo O.A., Capossela S., Bally L., Minder B., Brach M., Eriks-Hoogland I. (2021). Body Composition According to Spinal Cord Injury Level: A Systematic Review and Meta-Analysis. J. Clin. Med..

[89] Graupensperger S., Sweet S.N., Evans M.B. (2018). Multimorbidity of overweight and obesity alongside anxiety and depressive disorders in individuals with spinal cord injury. J. Spinal Cord Med..

[90] Wen H., Botticello A.L., Bae S., Heinemann A.W., Boninger M., Houlihan B.V., Chen Y. (2019). Racial and Ethnic Differences in Obesity in People with Spinal Cord Injury: The Effects of Disadvantaged Neighborhood. Arch. Phys. Med. Rehabil..

[91] Wen H., Chen Y., He Y., Bickel C.S., Robinson-Whelen S., Heinemann A.W. (2018). Racial Differences in Weight Gain: A 5-Year Longitudinal Study of Persons with Spinal Cord Injury. Arch. Phys. Med. Rehabil..

[92] DiPiro N.D., Murday D., Corley E.H., Krause J.S. (2018). Prevalence of chronic health conditions and hospital utilization in adults with spinal cord injury: An analysis of self-report and South Carolina administrative billing data. Spinal Cord.

[93] Cao Y., DiPiro N., Krause J.S. (2020). Association of Secondary Health Conditions with Future Chronic Health Conditions among Persons with Traumatic Spinal Cord Injury. Top. Spinal Cord Inj. Rehabil..

[94] South Carolina Department of Health and Environmental Control High Cholesterol. https://scdhec.gov/health/diseases-conditions/heart-disease-stroke/high-cholesterol.

[95] Tallqvist S., Kauppila A.M., Vainionpää A., Koskinen E., Bergman P., Anttila H., Hämäläinen H., Täckman A., Kallinen M., Arokoski J. (2021). Prevalence of comorbidities and secondary health conditions among the Finnish population with spinal cord injury. Spinal Cord.

[96] Laatikainen T., Tapanainen H., Jousilahti P., Valsta L., Vartiainen E. (2019). Suomalaisten Kolesterolitasot Ja Tyydyttyneen Rasvan Saanti Ylittävät Edelleen Suositukset.

[97] Walldius G., Jungner I., Kolar W., Holme I., Steiner E. (1992). High cholesterol and triglyceride values in Swedish males and females: Increased risk of fatal myocardial infarction. First report from the AMORIS (Apolipoprotein related MOrtality RISk) study. Blood Press. Suppl..

[98] Koyuncu E., Yüzer G.F.N., Yenigün D., Özgirgin N. (2016). The analysis of serum lipid levels in patients with spinal cord injury. J. Spinal Cord Med..

[99] Sabour H., Latifi S., Soltani Z., Shakeri H., Javidan A.N., Ghodsi S.-M., Hadian M.R., Razavi S.-H.E. (2016). C-reactive protein as an available biomarker determining mental component of health-related quality of life among individuals with spinal cord injury. J. Spinal Cord Med..

[100] La Fountaine M.F., Cirnigliaro C.M., Kirshblum S.C., McKenna C., Bauman W.A. (2017). Effect of functional sympathetic nervous system impairment of the liver and abdominal visceral adipose tissue on circulating triglyceride-rich lipoproteins. PLoS ONE.

[101] La Fountaine M.F., Cirnigliaro C.M., Hobson J.C., Dyson-Hudson T.A., Mc Kenna C., Kirshblum S.C., Spungen A.M., Bauman W.A. (2018). Establishing a threshold to predict risk of cardiovascular disease from the serum triglyceride and high-density lipoprotein concentrations in persons with spinal cord injury. Spinal Cord.

[102] Aidinoff E., Bluvshtein V., Bierman U., Gelernter I., Front L., Catz A. (2016). Coronary artery disease and hypertension in a non-selected spinal cord injury patient population. Spinal Cord.

[103] Adriaansen J.J.E., Douma-Haan Y., Van Asbeck F.W.A., Van Koppenhagen C.F., De Groot S., Smit C.A., Visser-Meily J.M.A., Post M.W.M. (2016). Allrisc Prevalence of hypertension and associated risk factors in people with long-term spinal cord injury living in the Netherlands. Disabil. Rehabil..

[104] Gater D.R., Farkas G.J., Berg A.S., Castillo C. (2019). Prevalence of metabolic syndrome in veterans with spinal cord injury. J. Spinal Cord Med..

[105] Ullah S., Qamar I., Qureshi A.Z., Abu-Shaheen A., Niaz A. (2018). Functional outcomes in geriatric patients with spinal cord injuries at a tertiary care rehabilitation hospital in Saudi Arabia. Spinal Cord Ser. Cases.

[106] Vriz O., Bertin N., Ius A., Bizzarini E., Bossone E., Antonini-Canterin F. (2017). Carotid artery stiffness and development of hypertension in people with paraplegia and no overt cardiovascular disease: A 7-year follow-up study. J. Cardiovasc. Echogr..

[107] Moussavi R.M., Ribas-Cardus F., Rintala D.H., Rodriguez G.P. (2001). Dietary and serum lipids in individuals with spinal cord injury living in the community. J. Rehabil. Res. Dev..

[108] Schelleman H., Klungel O.H., Kromhout D., De Boer A., Stricker B., Verschuren W.M.M. (2004). Prevalence and determinants of undertreatment of hypertension in the Netherlands. J. Hum. Hypertens..

[109] South Carolina Department of Health and Environmental Control (2018). State of the Heart Heart Disease in South Carolina.

[110] Al-Nozha M.M., Abdullah M., Arafah M.R., Khalil M.Z., Khan N.B., Al-Mazrou Y.Y., Al-Maatouq M.A., Al-Marzouki K., Al-Khadra A., Nouh M.S. (2007). Hypertension in Saudi Arabia. Saudi Med J..

[111] Koponen P., Reinikainen J., Tolonen H., Laatikainen T., Jousilahti P., Koskinen S. (2019). Prevalence of hypertension and diabetes in Finland by different data sources. Eur. J. Public Health.

[112] Swedish Council on Health Technology Assessment (2004). SBU Systematic Review Summaries. Moderately Elevated Blood Pressure: A Systematic Review.

[113] Groah S., Weitzenkamp D., Sett P., Soni B., Savic G. (2001). The relationship between neurological level of injury and symptomatic cardiovascular disease risk in the aging spinal injured. Spinal Cord.

[114] Hubli M., Gee C.M., Krassioukov A.V. (2014). Refined Assessment of Blood Pressure Instability After Spinal Cord Injury. Am. J. Hypertens..

[115] West C.R., Popok D., Crawford M.A., Krassioukov A.V. (2015). Characterizing the Temporal Development of Cardiovascular Dysfunction in Response to Spinal Cord Injury. J. Neurotrauma..

[116] Allen K.J., Leslie S.W. (2022). Autonomic Dysreflexia. StatPearls.

[117] Myers J., Lee M., Kiratli J. (2007). Cardiovascular Disease in Spinal Cord Injury. Am. J. Phys. Med. Rehabil..

[118] Li J., Hunter G.R., Chen Y., McLain A., Smith D.L., Yarar-Fisher C. (2018). Differences in Glucose Metabolism among Women with Spinal Cord Injury May Not Be Fully Explained by Variations in Body Composition. Arch. Phys. Med. Rehabil..

[119] State of the Heart Disease in South Carolina (2020). Diabetes Impact in South Carolina.

[120] Andersson T., Ahlbom A., Carlsson S. (2015). Diabetes Prevalence in Sweden at Present and Projections for Year. PLoS ONE.

[121] Naeem Z. (2015). Burden of Diabetes Mellitus in Saudi Arabia. Int. J. Health Sci..

[122] Chen Y., Wen H., Baidwan N.K., DeVivo M.J. (2022). Demographic and Health Profiles of People Living with Traumatic Spinal Cord Injury in the United States During 2015–2019: Findings from the Spinal Cord Injury Model Systems Database. Arch. Phys. Med. Rehabil..

[123] Mordarska K., Godziejewska-Zawada M. (2017). Diabetes in the elderly. Menopausal Rev..

[124] Kirkman M.S., Briscoe V.J., Clark N., Florez H., Haas L.B., Halter J.B., Huang E.S., Korytkowski M.T., Munshi M.N., Odegard P.S. (2012). Diabetes in Older Adults. Diabet. Care.

[125] Farkas G.J., Gordon P.S., Trewick N., Gorgey A.S., Dolbow D.R., Tiozzo E., Berg A.S., Gater D.R. (2021). Comparison of Various Indices in Identifying Insulin Resistance and Diabetes in Chronic Spinal Cord Injury. J. Clin. Med..

[126] Heymsfield S.B., Lichtman S., Baumgartner R.N., Wang J., Kamen Y., Aliprantis A., Pierson R.N. (1990). Body composition of humans: Comparison of two improved four-compartment models that differ in expense, technical complexity, and radiation exposure. Am. J. Clin. Nutr..

[127] Saklayen M.G. (2018). The Global Epidemic of the Metabolic Syndrome. Curr. Hypertens. Rep..

[128] Hirode G., Wong R.J. (2020). Trends in the Prevalence of Metabolic Syndrome in the United States, 2011–2016. JAMA.

[129] Kuk J.L., Ardern C.I., Church T.S., Sharma A.M., Padwal R., Sui X., Blair S.N. (2011). Edmonton Obesity Staging System: Association with weight history and mortality risk. Appl. Physiol. Nutr. Metab..

[130] Sharma A.M., Kushner R.F. (2009). A proposed clinical staging system for obesity. Int. J. Obes..

[131] Guo F., Garvey W.T. (2015). Development of a Weighted Cardiometabolic Disease Staging (CMDS) System for the Prediction of Future Diabetes. J. Clin. Endocrinol. Metab..

[132] Guo F., Moellering D.R., Garvey W.T. (2013). The progression of cardiometabolic disease: Validation of a new cardiometabolic disease staging system applicable to obesity. Obesity.

[133] D’Agostino R.B., Vasan R.S., Pencina M.J., Wolf P.A., Cobain M., Massaro J.M., Kannel W.B. (2008). General Cardiovascular Risk Profile for Use in Primary Care: The Framingham Heart Study. Circulation.

[134] Gander J., Sui X., Hazlett L.J., Cai B., Hébert J.R., Blair S.N. (2014). Factors Related to Coronary Heart Disease Risk among Men: Validation of the Framingham Risk Score. Prev. Chronic Dis..

[135] Centers for Disease Control and Prevention Heart Disease Facts. https://www.cdc.gov/heartdisease/facts.htm#:~:text=Heart%20disease%20is%20the%20leading,1%20in%20every%204%20deaths.

[136] Weaver F.M., Smith B., LaVela S.L., Evans C.T., Ullrich P., Miskevics S., Goldstein B., Strayer J., Burns S.P. (2011). Smoking behavior and delivery of evidence-based care for veterans with spinal cord injuries and disorders. J. Spinal Cord Med..

[137] Saunders L.L., Krause J.S., Carpenter M.J., Saladin M. (2013). Risk Behaviors Related to Cigarette Smoking among Persons with Spinal Cord Injury. Nicotine Tob. Res..

[138] Saunders L.L., Krause J.S., Saladin M., Carpenter M.J. (2015). Prevalence of cigarette smoking and attempts to quit in a population-based cohort with spinal cord injury. Spinal Cord.

[139] Ginis K.A.M., Latimer A., Arbour-Nicitopoulos K.P., Buchholz A.C., Bray S., Craven B., Hayes K.C., Hicks A.L., McColl M.A., Potter P.J. (2010). Leisure Time Physical Activity in a Population-Based Sample of People with Spinal Cord Injury Part I: Demographic and Injury-Related Correlates. Arch. Phys. Med. Rehabil..

[140] Verschuren O., Dekker B., van Koppenhagen C., Post M. (2016). Sedentary Behavior in People with Spinal Cord Injury. Arch. Phys. Med. Rehabil..

[141] Rauch A., Hinrichs T., Oberhauser C., Cieza A., for the SwiSCI Study Group (2015). Do people with spinal cord injury meet the WHO recommendations on physical activity?. Int. J. Public Health.

[142] Berg-Emons R.J.V.D., Bussmann J.B., Haisma J.A., Sluis T.A., van der Woude L., Bergen M.P., Stam H.J. (2008). A Prospective Study on Physical Activity Levels After Spinal Cord Injury During Inpatient Rehabilitation and the Year After Discharge. Arch. Phys. Med. Rehabil..

[143] Frisbie J.H., Tun C.G. (1984). Drinking and spinal cord injury. J. Am. Paraplegia Soc..

[144] Tate D.G., Forchheimer M., Krause J.S., Meade M.A., Bombardier C.H. (2004). Patterns of alcohol and substance use and abuse in persons with spinal cord injury: Risk factors and correlates. Arch. Phys. Med. Rehabil..

[145] Saunders L.L., Krause J.S. (2010). Psychological factors affecting alcohol use after spinal cord injury. Spinal Cord.

[146] Krause J.S., Kemp B., Coker J. (2000). Depression after spinal cord injury: Relation to gender, ethnicity, aging, and socioeconomic indicators. Arch. Phys. Med. Rehabil..

[147] Jorge A., White M., Agarwal N. (2018). Outcomes in socioeconomically disadvantaged patients with spinal cord injury: A systematic review. J. Neurosurg. Spine.

[148] Strauss D., DeVivo M., Shavelle R., Brooks J., Paculdo D. (2008). Economic Factors and Longevity in Spinal Cord Injury: A Reappraisal. Arch. Phys. Med. Rehabil..

[149] Krause J.S., DeVivo M.J., Jackson A.B. (2004). Health status, community integration, and economic risk factors for mortality after spinal cord injury. Arch. Phys. Med. Rehabil..

[150] Driussi C., Ius A., Bizzarini E., Antonini-Canterin F., D’Andrea A., Bossone E., Vriz O. (2014). Structural and functional left ventricular impairment in subjects with chronic spinal cord injury and no overt cardiovascular disease. J. Spinal Cord Med..

[151] Eysmann S.B., Douglas P.S., Katz S.E., Sarkarati M., Wei J.Y. (1995). Left ventricular mass and diastolic filling patterns in quadriplegia and implications for effects of normal aging on the heart. Am. J. Cardiol..

[152] Williams A.M., Gee C.M., Voss C., West C.R. (2018). Cardiac consequences of spinal cord injury: Systematic review and meta-analysis. Heart.

[153] Barton T.J., Low D.A., Bakker E.A., Janssen T., de Groot S., van der Woude L., Thijssen D.H. (2020). Traditional Cardiovascular Risk Factors Strongly Underestimate the 5-Year Occurrence of Cardiovascular Morbidity and Mortality in Spinal Cord Injured Individuals. Arch. Phys. Med. Rehabil..

[154] Wu J.-C., Chen Y.-C., Liu L., Chen T.-J., Huang W.-C., Cheng H., Tung-Ping S. (2012). Increased risk of stroke after spinal cord injury: A nationwide 4-year follow-up cohort study. Neurology.

[155] Miyatani M., Alavinia M., Szeto M., Moore C., Craven B. (2017). Association between abnormal arterial stiffness and cardiovascular risk factors in people with chronic spinal cord injury. Eur. J. Prev. Cardiol..

[156] Currie K.D., Hubli M., Macdonald M.J., Krassioukov A.V. (2019). Associations between arterial stiffness and blood pressure fluctuations after spinal cord injury. Spinal Cord.

[157] Wahman K., Nash M., Lewis J., Seiger Å., Levi R. (2011). Cardiovascular disease risk and the need for prevention after paraplegia determined by conventional multifactorial risk models: The Stockholm spinal cord injury study. J. Rehabil. Med..

[158] Wahman K., Nash M., Lewis J., Seiger Å., Levi R. (2010). Increased cardiovascular disease risk in Swedish persons with paraplegia: The Stockholm spinal cord injury study. J. Rehabil. Med..

[159] Wilt T.J., Carlson K.F., Goldish G.D., MacDonald R., Niewoehner C., Rutks I., Shamliyan T., Tacklind J., Taylor B.C., Kane R.L. (2008). Carbohydrate and lipid disorders and relevant considerations in persons with spinal cord injury. Évid. Rep. Assess..

[160] Roth G.A., Mensah G.A., Johnson C.O., Addolorato G., Ammirati E., Baddour L.M., Barengo N.C., Beaton A.Z., Benjamin E.J., Benziger C.P. (2020). Global Burden of Cardiovascular Diseases and Risk Factors, 1990–2019: Update From the GBD 2019 Study. J. Am. Coll. Cardiol..

[161] Afshin A., Sur P.J., Fay K.A., Cornaby L., Ferrara G., Salama J.S., Mullany E.C., Abate K.H., Abbafati C., Abebe Z. (2019). Health effects of dietary risks in 195 countries, 1990–2017: A systematic analysis for the Global Burden of Disease Study 2017. Lancet.

[162] Froehlich-Grobe K., Nary D.E., VanSciver A., Washburn R.A., Aaronson L. (2012). Truth Be Told: Evidence of Wheelchair Users’ Accuracy in Reporting Their Height and Weight. Arch. Phys. Med. Rehabil..

[163] Froehlich-Grobe K., Nary D.E., Van Sciver A., Lee J., Little T.D. (2011). Measuring Height without a Stadiometer. Am. J. Phys. Med. Rehabilitation.

[164] Bray G.A. (1996). HEALTH HAZARDS OF OBESITY. Endocrinol. Metab. Clin. N. Am..

[165] Cole T., Himes J. (1991). Weight-stature indices to measure underweight, overweight, and obesity. Anthropometric Assessment of Nutritional Status.

[166] Gallagher D., Heymsfield S.B., Heo M., Jebb S.A., Murgatroyd P.R., Sakamoto Y. (2000). Healthy percentage body fat ranges: An approach for developing guidelines based on body mass index. Am. J. Clin. Nutr..

[167] Farkas G.J., Swartz A.M., Gorgey A.S., Berg A.S., Gater D.R. (2021). Acute exercise improves glucose effectiveness but not insulin sensitivity in paraplegia. Disabil. Rehabil..

[168] Farkas G.J., Gorgey A.S., Dolbow D.R., Berg A.S., Gater D.R. (2021). Energy Expenditure, Cardiorespiratory Fitness, and Body Composition Following Arm Cycling or Functional Electrical Stimulation Exercises in Spinal Cord Injury: A 16-Week Randomized Controlled Trial. Top. Spinal Cord Inj. Rehabil..

[169] Ma Y., de Groot S., Weijs P.J.M., Achterberg W., Adriaansen J., Janssen T.W.J. (2021). Accuracy of bioelectrical impedance analysis and skinfold thickness in the assessment of body composition in people with chronic spinal cord injury. Spinal Cord.

[170] Bigford G.E., Mendez A.J., Betancourt L., Burns-Drecq P., Backus D., Nash M.S. (2017). A lifestyle intervention program for successfully addressing major cardiometabolic risks in persons with SCI: A three-subject case series. Spinal Cord Ser. Cases.

[171] Dolbow D.R., Credeur D.P., Lemacks J.L., Stokic D.S., Pattanaik S., Corbin G.N., Courtner A.S. (2020). Electrically induced cycling and nutritional counseling for counteracting obesity after spinal cord injury: A pilot study. J. Spinal Cord Med..

[172] Jacobs P.L., Mahoney E.T., Nash M.S., Green B.A. (2002). Circuit resistance training in persons with complete paraplegia. J. Rehabilitation Res. Dev..

[173] Nash M.S., Jacobs P.L., Mendez A.J., Goldberg R.B. (2001). Circuit resistance training improves the atherogenic lipid profiles of persons with chronic paraplegia. J. Spinal Cord Med..

[174] Nash M.S., van de Ven I., van Elk N., Johnson B.M. (2007). Effects of Circuit Resistance Training on Fitness Attributes and Upper-Extremity Pain in Middle-Aged Men with Paraplegia. Arch. Phys. Med. Rehabil..

[175] Nash M.S., Kressler J. (2016). Model Programs to Address Obesity and Cardiometabolic Disease: Interventions for Suboptimal Nutrition and Sedentary Lifestyles. Arch. Phys. Med. Rehabil..

[176] Farrow M., Nightingale T., Maher J., McKay C.D., Thompson D., Bilzon J.L. (2020). Effect of Exercise on Cardiometabolic Risk Factors in Adults with Chronic Spinal Cord Injury: A Systematic Review. Arch. Phys. Med. Rehabil..

[177] Bakkum A., Paulson T., Bishop N., Goosey-Tolfrey V., Stolwijk-Swã¼Ste J., Kuppevelt D., Groot S., Janssen T. (2015). Effects of hybrid cycle and handcycle exercise on cardiovascular disease risk factors in people with spinal cord injury: A randomized controlled trial. J. Rehabil. Med..

[178] de Zepetnek J.O.T., Pelletier C.A., Hicks A.L., MacDonald M.J. (2015). Following the Physical Activity Guidelines for Adults with Spinal Cord Injury for 16 Weeks Does Not Improve Vascular Health: A Randomized Controlled Trial. Arch. Phys. Med. Rehabil..

[179] Kim D.-I., Taylor J.A., Tan C.O., Park H., Kim J.Y., Park S.-Y., Chung K.-M., Lee Y.-H., Lee B.-S., Jeon J.Y. (2019). A pilot randomized controlled trial of 6-week combined exercise program on fasting insulin and fitness levels in individuals with spinal cord injury. Eur. Spine J..

[180] Nightingale T.E., Walhin J.-P., Thompson D., Bilzon J.L.J. (2017). Impact of Exercise on Cardiometabolic Component Risks in Spinal Cord–injured Humans. Med. Sci. Sports Exerc..

[181] Bresnahan J.J., Farkas G.J., Clasey J.L., Yates J.W., Gater D.R. (2017). Arm crank ergometry improves cardiovascular disease risk factors and community mobility independent of body composition in high motor complete spinal cord injury. J. Spinal Cord Med..

[182] Kim D.-I., Lee H., Lee B.-S., Kim J., Jeon J.Y. (2015). Effects of a 6-Week Indoor Hand-Bike Exercise Program on Health and Fitness Levels in People with Spinal Cord Injury: A Randomized Controlled Trial Study. Arch. Phys. Med. Rehabil..

[183] Li J., Polston K.F.L., Eraslan M., Bickel C.S., Windham S.T., McLain A.B., Oster R.A., Bamman M.M., Yarar-Fisher C. (2018). A high-protein diet or combination exercise training to improve metabolic health in individuals with long-standing spinal cord injury: A pilot randomized study. Physiol. Rep..

[184] Graham K., Yarar-Fisher C., Li J., McCully K.M., Rimmer J.H., Powell D., Bickel C.S., Fisher G. (2019). Effects of High-Intensity Interval Training Versus Moderate-Intensity Training on Cardiometabolic Health Markers in Individuals with Spinal Cord Injury: A Pilot Study. Top. Spinal Cord Inj. Rehabil..

[185] Mogharnasi M., TaheriChadorneshin H., Papoli-Baravati S.A., Teymuri A. (2019). Effects of upper-body resistance exercise training on serum nesfatin-1 level, insulin resistance, and body composition in obese paraplegic men. Disabil. Health J..

[186] Horiuchi M., Okita K. (2017). Arm-Cranking Exercise Training Reduces Plasminogen Activator Inhibitor 1 in People with Spinal Cord Injury. Arch. Phys. Med. Rehabil..

[187] Cugusi L., Solla P., Serpe R., Pilia K., Pintus V., Madeddu C., Bassareo P., Mercuro G. (2015). Effects of an adapted physical training on functional status, body composition and quality of life in persons with spinal cord injury paraplegia: A pilot study. Med. Sport.

[188] Bochkezanian V., Newton R.U., Trajano G.S., Blazevich A.J. (2018). Effects of Neuromuscular Electrical Stimulation in People with Spinal Cord Injury. Med. Sci. Sports Exerc..

[189] Ginis K.A.M., Van Der Scheer J.W., Latimer A., Barrow A., Bourne C., Carruthers P., Bernardi M., Ditor D.S., Gaudet S., De Groot S. (2017). Evidence-based scientific exercise guidelines for adults with spinal cord injury: An update and a new guideline. Spinal Cord.

[190] Haisma J.A., Van Der Woude L.H.V., Stam H.J., Bergen M.P., Sluis T.A.R., Bussmann J.B.J. (2006). Physical capacity in wheelchair-dependent persons with a spinal cord injury: A critical review of the literature. Spinal Cord.

[191] Berg-Emons R.J.V.D., Bussmann J.B., Stam H.J. (2010). Accelerometry-Based Activity Spectrum in Persons with Chronic Physical Conditions. Arch. Phys. Med. Rehabil..

[192] Scelza W.M., Kalpakjian C.Z., Zemper E.D., Tate D.G. (2005). Perceived Barriers to Exercise in People with Spinal Cord Injury. Am. J. Phys. Med. Rehabil..

[193] Kroll T., Kratz A., Kehn M., Jensen M.P., Groah S., Ljungberg I.H., Molton I.R., Bombardier C. (2012). Perceived Exercise Self-efficacy as a Predictor of Exercise Behavior in Individuals Aging with Spinal Cord Injury. Am. J. Phys. Med. Rehabil..

[194] Cowan R., Nash M.S., Anderson-Erisman K. (2012). Perceived Exercise Barriers and Odds of Exercise Participation among Persons with SCI Living in High-Income Households. Top. Spinal Cord Inj. Rehabil..

[195] Cowan R., Nash M.S., Anderson K.D. (2012). Exercise participation barrier prevalence and association with exercise participation status in individuals with spinal cord injury. Spinal Cord.

[196] Noreau L., Shephard R.J., Simard C., Paré G., Pomerleau P. (1993). Relationship of impairment and functional ability to habitual activity and fitness following spinal cord injury. Int. J. Rehabil. Res..

[197] Boninger M.L., Dicianno B.E., Cooper R.A., Towers J.D., Koontz A.M., Souza A.L. (2003). Shoulder magnetic resonance imaging abnormalities, wheelchair propulsion, and gender. Arch. Phys. Med. Rehabil..

[198] Alvarado J.R.V., Felix E.R., Gater D.R. (2021). Upper Extremity Overuse Injuries and Obesity after Spinal Cord Injury. Top. Spinal Cord Inj. Rehabil..

[199] Ballinger D.A., Rintala D.H., Hart K.A. (2000). The relation of shoulder pain and range-of-motion problems to functional limitations, disability, and perceived health of men with spinal cord injury: A multifaceted longitudinal study. Arch. Phys. Med. Rehabil..

[200] Van Hall G., Jensen-Urstad M., Rosdahl H., Holmberg H.-C., Saltin B., Calbet J.A. (2003). Leg and arm lactate and substrate kinetics during exercise. Am. J. Physiol. Metab..

[201] McMillan D.W., Maher J.L., Jacobs K.A., Nash M.S., Gater D.R. (2021). Exercise Interventions Targeting Obesity in Persons with Spinal Cord Injury. Top. Spinal Cord Inj. Rehabil..

[202] Gorgey A.S., Dolbow D.R., Dolbow J.D., Khalil R.K., Gater D.R. (2014). The effects of electrical stimulation on body composition and metabolic profile after spinal cord injury—Part II. J. Spinal Cord Med..

[203] Ragnarsson K.T. (2007). Functional electrical stimulation after spinal cord injury: Current use, therapeutic effects and future directions. Spinal Cord.

[204] Ragnarsson K.T. (1988). Physiologic effects of functional electrical stimulation-induced exercises in spinal cord-injured individuals. Clin. Orthop. Relat. Res..

[205] Rodgers M.M., Glaser R.M., Figoni S.E., Hooker S., Ezenwa B.N., Collins S.R., Mathews T., Suryaprasad A.G., Gupta S.C. (1991). Musculoskeletal responses of spinal cord injured individuals to functional neuromuscular stimulation-induced knee extension exercise training. J. Rehabil. Res. Dev..

[206] Hooker S.P., Wells C.L. (1989). Effects of low-and moderate-intensity training in spinal cord-injured persons. Med. Sci. Sports Exerc..

[207] Gorgey A.S., Harnish C.R., Daniels J.A., Dolbow D.R., Keeley A., Moore J., Gater D.R. (2012). A report of anticipated benefits of functional electrical stimulation after spinal cord injury. J. Spinal Cord Med..

[208] Hjeltnes N., Galuska D., Björnholm M., Aksnes A.-K., Lannem A., Zierath J., Wallberg-Henriksson H. (1998). Exercise-induced overexpression of key regulatory proteins involved in glucose uptake and metabolism in tetraplegic persons: Molecular mechanism for improved glucose homeostasis. FASEB J..

[209] Figoni S.F., Rodgers M.M., Glaser R.M., Hooker S.P., Feghri P.D., Ezenwa B.N., Mathews T., Suryaprasad A.G., Gupta S.C. (1990). Physiologic Responses of Paraplegics and Quadriplegics to Passive and Active Leg Cycle Ergometry. J. Am. Paraplegia Soc..

[210] Arnold P.B., McVey P.P., Farrell W.J., Deurloo T.M., Grasso A.R. (1992). Functional electric stimulation: Its efficacy and safety in improving pulmonary function and musculoskeletal fitness. Arch. Phys. Med. Rehabil..

[211] Barstow T.J., Scremin A.M.E., Mutton D.L., Kunkel C.F., Cagle T.G., Whipp B.J. (1996). Changes in gas exchange kinetics with training in patients with spinal cord injury. Med. Sci. Sports Exerc..

[212] Nash M.S., Bilsker M.S., Kearney H.M., Ramirez J.N., Applegate B., Green B.A. (1995). Effects of electrically-stimulated exercise and passive motion on echocardiographically-derived wall motion and cardiodynamic functic in tetraplegic persons. Spinal Cord.

[213] Griffin L., Decker M., Hwang J., Wang B., Kitchen K., Ding Z., Ivy J. (2009). Functional electrical stimulation cycling improves body composition, metabolic and neural factors in persons with spinal cord injury. J. Electromyogr. Kinesiol..

[214] Rosety-Rodriguez M., Camacho A., Rosety I., Fornieles G., Rosety M.A., Diaz A.J., Bernardi M., Rosety M., Ordonez F.J. (2013). Low-Grade Systemic Inflammation and Leptin Levels Were Improved by Arm Cranking Exercise in Adults with Chronic Spinal Cord Injury. Arch. Phys. Med. Rehabil..

[215] Bigford G., Nash M.S. (2017). Nutritional Health Considerations for Persons with Spinal Cord Injury. Top. Spinal Cord Inj. Rehabil..

[216] Chen Y., Henson S.L., Jackson A.B., Richards J.S. (2005). Obesity intervention in persons with spinal cord injury. Spinal Cord.

[217] Allison D.J., Beaudry K.M., Thomas A.M., Josse A., Ditor D.S. (2018). Changes in nutrient intake and inflammation following an anti-inflammatory diet in spinal cord injury. J. Spinal Cord Med..

[218] Nash M.S., Groah S.L., Gater D.R., Dyson-Hudson T., Lieberman J.A., Myers J., Sabharwal S., Taylor A.J. (2018). Consortium for Spinal Cord Medicine Identification and Management of Cardiometabolic Risk after Spinal Cord Injury: Clinical Practice Guideline for Health Care Providers. Top. Spinal Cord Inj. Rehabil..

[219] U.S. Department of Health and Human Services and U.S. Department of Agriculture (2020). 2020–2025 Dietary Guidelines for Americans.

[220] Nightingale T.E., Gorgey A.S. (2018). Predicting Basal Metabolic Rate in Men with Motor Complete Spinal Cord Injury. Med. Sci. Sports Exerc..

[221] Chun S.M., Kim H.-R., Shin H.I. (2017). Estimating the Basal metabolic rate from fat free mass in individuals with motor complete spinal cord injury. Spinal Cord.

[222] Buchholz A.C., McGillivray C., Pencharz P.B. (2003). Differences in resting metabolic rate between paraplegic and able-bodied subjects are explained by differences in body composition. Am. J. Clin. Nutr..

[223] Kelly H. (2011). The classical definition of a pandemic is not elusive. Bull. World Health Organ..

